# Quantifying Gas Adsorption Variability and Optimal Si/Al Ratio for Rational Design of Aluminum-Substituted Zeolite Frameworks

**DOI:** 10.1021/acs.langmuir.5c00276

**Published:** 2025-06-11

**Authors:** Akhilesh Gandhi, Silabrata Pahari, Joseph Sang-II Kwon, M. M. Faruque Hasan

**Affiliations:** † Artie McFerrin Department of Chemical Engineering, 14736 Texas A&M University, College Station, Texas 77843-3122, United States; ‡ Texas A&M Energy Institute, 2655 Texas A&M University, College Station, Texas 77843, United States

## Abstract

Experimental measurements often show significant variations in gas adsorption on aluminosilicate zeolites, thereby inducing considerable uncertainty in the gas separation and storage performance. These variations are largely attributed to the distribution of aluminum (Al) atoms within the zeolite framework. It is challenging to experimentally control the distribution of Al atoms during zeolite synthesis. The vast number of plausible Al-substituted configurations also makes it difficult to estimate the overall range of adsorption. To resolve this, we deploy a new representation of crystallographic frameworks using single repeating units (SRU). An SRU consists of the smallest network of tetrahedral atoms that can be repeated as a single building block to represent an entire zeolite framework. SRUs enable a selective enumeration of unique Al-substituted configurations, thereby leading to an efficient computational framework for quantifying the variations in equilibrium gas adsorption on Al-substituted zeolites without an exhaustive search. We apply this technique to analyze CO_2_ adsorption on chabazite (CHA) zeolite. Using molecular simulations of gas adsorption on the unique Al-substituted configurations, we observe as much as 12% variation in the CO_2_ adsorption due to differences in the locations of Al atoms within the zeolite framework. Interestingly, our results indicate that variability in CO_2_ adsorption in Al-substituted zeolites is significant only at moderate Si/Al ratios, primarily due to the nonuniform distribution of Al. At very high or very low Si/Al ratios, this variability appears to be negligible. Surprisingly, we also observe that the adsorption does not always increase with the number of Al sites, and there exists an inflection point beyond which additional Al substitution leads to a decrease in adsorption. This trade-off indicates an optimal Si/Al ratio that maximizes the equilibrium adsorption of CO_2_ on Al-substituted CHA zeolites at some moderate values. We are able to systematically identify the optimal Si/Al ratio and the corresponding locations of Al sites in the CHA framework that maximizes CO_2_ adsorption. On further investigation using the Al–Al radial distribution function (RDF), we find the locations of Al sites that lead to high CO_2_ adsorption. This demonstrates that the SRU-based selective enumeration combined with RDF-based structural screening is an enabling method toward the rational design of zeolites with optimal distribution of Al sites to achieve desired properties.

## Graphical abstract

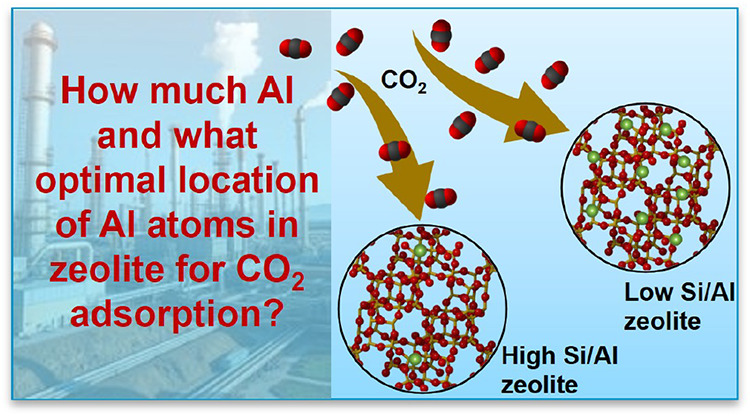

## 1 Introduction

Zeolites are aluminosilicate materials with a wide range of applications in gas separation, catalysis, ion exchange, and other fields.^1–5^ More than 250 zeolites have been naturally found or chemically synthesized, and millions of hypothetical structures have been analyzed using computer-based techniques.^6^ Like most aluminosilicates, zeolite frameworks are formed by aluminum (Al) and silicon (Si) atoms through the formation of Si–O–Al, Si–O–Si, and Al–O–Al oxide linkages. These linkages lead to a network of tetrahedral atoms (T-sites) to give rise to a 3D porous structure. The distribution and spatial arrangement of Al atoms within a zeolite framework particularly affect the adsorption of various gases within the framework. Experimental measurements have indicated considerable differences in equilibrium gas adsorption due to Al distribution within a zeolite framework. Up to two-fold differences in adsorption capacities of *n*-alkanes have been observed, for example, over Bronsted acid zeolites with the same Si/Al ratio.^7^ Conventional wisdom is that Al substitutions generally increase gas adsorption.^8^ However, this may not be true for all cases. Recent studies^9,10^ on small-pore zeolite frameworks, such as Gismondine (GIS) and Merlinoite (MER), have revealed the existence of optimal Si/Al ratio beyond which adsorption decreases with additional Al substitution. Data have also been reported^11^ where CO_2_ adsorption increases with increasing Al substitution until a plateau or peak is reached, beyond which no significant increase in adsorption is observed.

The mechanisms underlying adsorption on Al-substituted zeolites are complex and require further investigation. For fixed pore size and basicity, the electric field strength generated by cations through Al substitution influences the gas adsorption in microporous crystalline materials.^12^ The siting and orientation of Al atoms at important T-sites as well as the proximity of Al atoms to each other influence the force field within the lattice and determine the overall adsorption capacity. When Al atoms are strategically located at specific T-sites, they can synergistically enhance adsorption, leading to higher adsorption and selectivity for certain gases. Dispersion interactions also play an important role. For example, dispersion interactions account for about 50% of the overall adsorption enthalpy of CO_2_ molecules in FAU zeolite with a Si/Al ratio of 2.55:1.^13^ Guest CO_2_ molecules predominantly adsorb on site II and tilt toward the zeolite wall due to stabilizing dispersion interactions, with minor heterogeneity in adsorption sites arising from differences in the number of Al atoms and the geometry affecting their spatial arrangement. Other factors, such as the cation type, size, and charge, and the Si/Al ratio also affect the equilibrium and kinetic properties of gas adsorption on zeolites.^14–16^

It is important to accurately estimate the gas adsorption properties of zeolites to be able to design feasible chemical processes with desired separation and/or catalytic performance.^17–23^ To this end, the following questions remain unanswered: How do the location and configuration of Al substitutions affect the adsorption capacity of a zeolite? What is the optimal Si/Al ratio that maximizes gas adsorption, and how can we systematically identify this ratio?

The absence of literature regarding variation in adsorption on Al-substituted zeolites is primarily due to the challenges associated with comprehensive experimental and computational measurements and observations. There are recent successes in experimentally biasing the arrangement of Al and acid sites,^24–29^ but experimental synthesis of samples with precise control over each Al-siting and distribution is still difficult. Computational methods, on the other hand, typically rely on time-consuming and resource-intensive molecular simulations.^30^ These calculations may range on the order of days to weeks to obtain a single adsorption estimate on a single zeolite. While AI/ML-based predictive models are being developed,^31,32^ high-throughput screening of Al-substituted zeolites remains computationally demanding, given the large number of possible framework structures. The complexity further increases when dopants, such as Al or other metals, are used to replace Si in zeolite frameworks.^33^ Recent studies have demonstrated significant advancements in the development of quantitative structure–property relationships, driven by the emergence of advanced machine learning architectures such as Transformers.^34–37^ These data-dependent approaches often lack a systematic understanding grounded in first principles. Moving forward, it is essential to establish effective screening rules while maintaining a deeper understanding of the underlying system.^38^

The most challenging issue in studying the variability in adsorption of Al-substituted zeolites is probably the vast number of possible Al-substituted configurations. To illustrate this, consider the CHA zeolite framework, which has 36 T-sites in each unit cell. For a Si/Al ratio of 5.0, it would require substituting 6 of the 36 sites with Al. Without considering additional constraints, such substitutions can be achieved in $\left(\begin{array}{l} 36\\ 6 \end{array}\right)$ ways, leading to over 1.94 million possible structure configurations to check. As the number of Al atoms in the CHA framework increases, the number of possible Al-substituted configurations increases. For the Si/Al ratio of 1.0, which has 18 Al atoms and 18 Si atoms in a unit cell, this increases to over 9 million configurations. Even after limiting to only feasible structures via Löwenstein’s rule, which forbids Al–O–Al pairs in zeolites, the total number of plausible zeolite configurations is overwhelming. The enumeration of Al-substituted structures adds additional complexity to already time-intensive molecular simulations. No systematic approach currently exists to address this highly combinatorially complex problem, which seems to be daunting, even for a fixed Si/Al ratio. To the best of our knowledge, a systematic study investigating the effect of Al-siting on gas adsorption has also not yet been performed.

In this work, we overcome these combinatorial challenges in assessing the unique Al-substituted structures through exploiting a new graph-theoretic representation of zeolite frameworks using Single Repeating Units (SRU). Through SRUs, we can place Al atoms in unique sites while complying with Lowenstein’s rule, thereby allowing us to systematically enumerate only the unique Al-substituted configurations for a given Si/Al ratio, while representing most of the variations in Al sites. This is a key contribution of this work, as it significantly reduces the number of configurations needed for analysis and leads to a very efficient computational framework, which is described in detail in Section 2, for quantifying the variability observed in equilibrium gas adsorption, storage, and other properties of Al-substituted zeolites.

The paper is structured as follows: Section 2 outlines the methodology, including the SRU-based enumeration and generation of unique Al-substituted zeolite structure configurations and molecular simulation to obtain gas adsorption. In Section 3.1, we quantify the variation in CO_2_ adsorption on Al-substituted SOD and CHA zeolite frameworks for different Si/Al ratios, and systematically analyze the effects of proximity and location of distributed Al sites on CO_2_ adsorption. In Section 3.2, we propose a new approach, based on the similarity in the radial distribution function (RDF) and the CO_2_ adsorption, for high-throughput screening of optimal locations of Al sites in the lattice of the Al-substituted CHA zeolite framework that maximize the CO_2_ adsorption. We provide our concluding remarks in Section 4.

## 2 Methods

Figure 1 provides an overview of the workflow of the computational framework for the enumeration and analysis of uniquely Al-substituted zeolite configurations. It begins by taking the crystallographic information file (CIF) of the zeolite of interest. CIF is a standard way of describing a crystal framework. With the CIF data available, the first major step is to obtain the SRU representation of the zeolite structure. An SRU is the single smallest network of T-sites with defined connectivity that can be repeated to represent an entire zeolite framework.^39^ We have several efficient techniques/algorithms to identify the smallest set of T-nodes and their connectivity matrix that define the SRU of a zeolite framework (see Section 2.1 for SRU identification). We then use the SRU representation to generate structures with the desired number of Al substitutions, while maintaining a user-specified, fixed Si/Al ratio. As a check, the algorithm generates the postsubstitution connectivity matrices and determines their feasibility according to Lowenstein’s rule. Once the algorithm determines the feasible Al-substituted connectivity matrices, we use them to create the lattices and the unit cells needed to generate the individual CIF files for Al-substituted zeolite frameworks (see Section 2.2 for the generation of unique Al-substituted structures). We provide the new CIF files as inputs to a molecular simulation platform to predict the equilibrium adsorption of guest molecules, such as CO_2_, on different Al-substituted zeolite configurations for the same Si/Al ratio. We analyze the simulation data and quantify the variations in gas adsorption due to variations in Al substitution within a zeolite framework. We repeat the above procedure by parametrically changing the number of Al atoms within a zeolite framework. Overall, the SRU-based selective enumeration of Al-substituted structures enables a rational design of aluminosilicate zeolites with optimal Si/Al ratio to achieve desired gas adsorption and storage properties.

**Figure 1:**
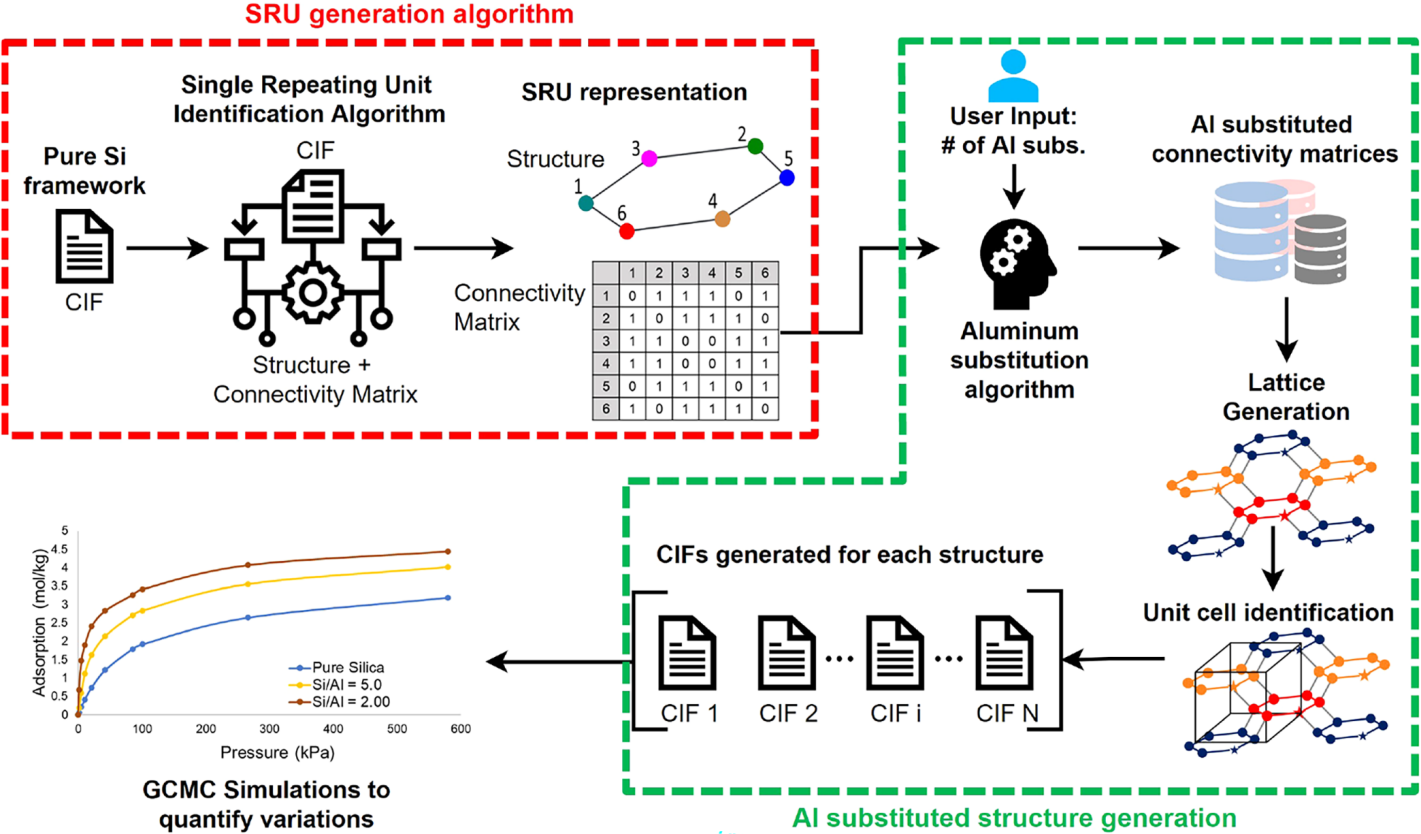
Overview of the workflow of the proposed computational framework, describing the enumeration and analysis of uniquely Al-substituted zeolite configurations. An efficient subset selection of unique distributions of Al for a given Si/Al ratio is enabled by the SRU (single repeating unit)-based representation of zeolite frameworks. An SRU is the smallest network of T-sites that can be repeated to generate an entire zeolite framework. It is used to generate uniquely Al-substituted structures. GCMC simulations are then performed on select Al-distributed structures to quantify the adsorption capacities.

### 2.1 SRU Identification

The SRU representation of zeolite frameworks was first proposed in 2021.^39,40^ An SRU consists of a structure that has fewer T-nodes than the unit cell and a connectivity matrix that governs the rules of connectivity between SRUs for regenerating the lattice. This graph-theoretic representation via the connectivity matrix is unique with the benefit of enumerating structural rules based on connectivity.

The graph-theoretic SRU representation enables the systematic enumeration of Al-substituted zeolite structures following Löwenstein’s rule. For example, the CHA SRU has only 12 T atoms and thus only 66 structures can be enumerated with a Si/Al ratio of 5.00 using the expression $\left(\begin{array}{l} 12\\ 2 \end{array}\right)$ . Using the SRU representation, a much larger number of structures can be efficiently generated and evaluated, enabling a better understanding of the impact of Si/Al substitution on the zeolite properties and accelerating the discovery of new materials for various applications.

The benefit of the SRU representation lies in its reduced representation compared with the traditional unit cell representation. To demonstrate this, we consider the CHA unit cell as a reference, which requires 3 CHA-SRU units to represent. If we want to replicate all 1.9 million possibilities that the unit cell offers, we can choose SRUs to get the desired Si/Al ratio in the unit cell and then identify different combinations of these SRUs. Once we generate these possibilities, we need to filter through all the 1.9 million structures based on Löwenstein’s rule. Table 1 shows the calculations based on the smaller structure (12-member unit similar to SRU without the graph representation) without exploiting the benefits of the graph-theoretic representation. This table shows the possibilities for the reconstruction of unit cell enumeration where each column shows the possible number of structures for the selected Al substitution. In the table, we calculate the total number of ways of selecting which smaller units to use and unique placements of the same which lead to a total of 1.9 million, equal to $\left(\begin{array}{l} 36\\ 6 \end{array}\right)$ .

**Table 1:** Possibilities for Reconstruction of Unit Cell Enumeration with Graph Theory Not Utilized for CHA with a Si/Al Ratio of 5.00

# subs. in SRU	0	1	2	3	4	5	6			
Possibilities	$\left(\begin{array}{l} 12\\ 0 \end{array}\right)$	$\left(\begin{array}{l} 12\\ 1 \end{array}\right)$	$\left(\begin{array}{l} 12\\ 2 \end{array}\right)$	$\left(\begin{array}{l} 12\\ 3 \end{array}\right)$	$\left(\begin{array}{l} 12\\ 4 \end{array}\right)$	$\left(\begin{array}{l} 12\\ 5 \end{array}\right)$	$\left(\begin{array}{l} 12\\ 6 \end{array}\right)$	Select	Orientations	Total
	2						1	$\left(\begin{array}{l} 12\\ 6 \end{array}\right)\left(\begin{array}{l} 12\\ 0 \end{array}\right)\left(\begin{array}{l} 12\\ 0 \end{array}\right)$	$\frac{3!}{2!1!}$	2772
	1	1				1		$\left(\begin{array}{l} 12\\ 5 \end{array}\right)\left(\begin{array}{l} 12\\ 1 \end{array}\right)\left(\begin{array}{l} 12\\ 0 \end{array}\right)$	$\frac{3!}{1!1!1!}$	57,024
	1		1		1			$\left(\begin{array}{l} 12\\ 4 \end{array}\right)\left(\begin{array}{l} 12\\ 2 \end{array}\right)\left(\begin{array}{l} 12\\ 0 \end{array}\right)$	$\frac{3!}{1!1!1!}$	196,020
		2			1			$\left(\begin{array}{l} 12\\ 4 \end{array}\right)\left(\begin{array}{l} 12\\ 1 \end{array}\right)\left(\begin{array}{l} 12\\ 1 \end{array}\right)$	$\frac{3!}{2!1!}$	213,840
	1			2				$\left(\begin{array}{l} 12\\ 3 \end{array}\right)\left(\begin{array}{l} 12\\ 3 \end{array}\right)\left(\begin{array}{l} 12\\ 0 \end{array}\right)$	$\frac{3!}{2!1!}$	145,200
		1	1	1				$\left(\begin{array}{l} 12\\ 3 \end{array}\right)\left(\begin{array}{l} 12\\ 2 \end{array}\right)\left(\begin{array}{l} 12\\ 1 \end{array}\right)$	$\frac{3!}{1!1!1!}$	1,045,440
			3					$\left(\begin{array}{l} 12\\ 2 \end{array}\right)\left(\begin{array}{l} 12\\ 2 \end{array}\right)\left(\begin{array}{l} 12\\ 2 \end{array}\right)$	$\frac{3!}{3!}$	287,496
									Total	1,947,792

In Table 2, we demonstrate the reduced number of structures generated by using the methodology proposed in this paper. The select column in this table refers to the possible selections of the selected substitutions, and details on how these numbers are obtained are discussed partially in a previous work.^39^ Note that the difference here is the number of possibilities for each level of substitution, and details of how the number of possible structures is reduced will be covered in the methodology using the node index method. There is 1 order of magnitude reduction in the total number of structures that need to be considered. However, given that the number is still significantly high in the computational requirements, we make the assumption of the periodicity of the lattice. We assume that the lattice is periodic over the SRU size. In the case of the unit cell, the periodicity is repeated over the unit cell, and thus, this assumption is in line with assuming periodicity in the smallest representative unit considered. There are some consequences to this assumption that some high Si/Al ratio structures may not be covered; however, that is the case for any baseline structure that can be arbitrarily chosen and some structures will still be excluded. Figure 2 provides a visual demonstration of the reduction in possible structures due to the use of the SRU in contrast to the unit cell.

**Figure 2:**
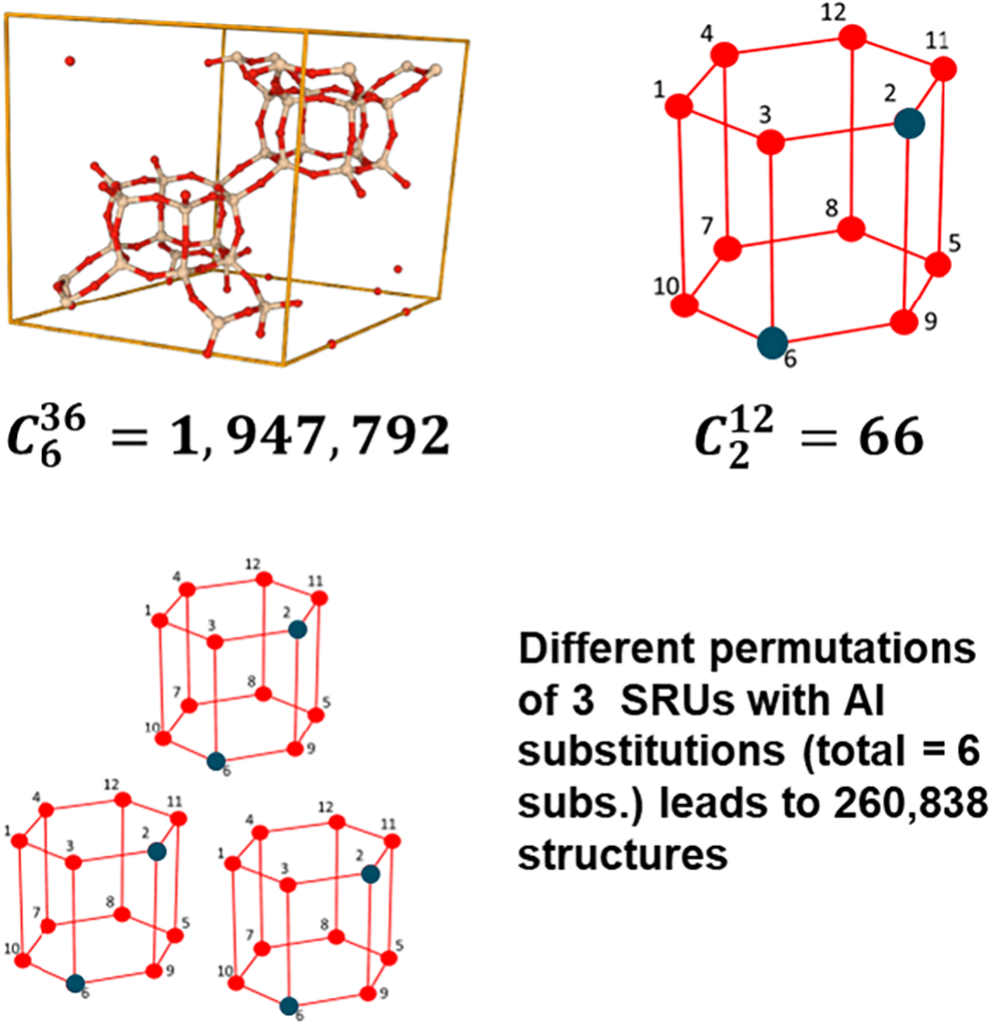
Strategic reduction in total possible Al-substituted structure by using SRU and permutations and combinations. The total Al substitutions in the unit cell still remain at 6.

**Table 2:** Possibilities for the Reconstruction of Unit Cell from SRU with the Benefit of Graph-Theoretic Representation for CHA with a Si/Al Ratio of 5.00

# subs. in SRU	0	1	2	3	4	5	6			
Possibilities	1	12	42	52	30	12	2	Select	Orientations	Total
	2						1	2	$\frac{3!}{2!1!}$	6
	1	1				1		12*12	$\frac{3!}{1!1!1!}$	864
	1		1		1			30*42	$\frac{3!}{1!1!1!}$	7560
		2			1			30*12*12	$\frac{3!}{2!1!}$	12,960
	1			2				52*52	$\frac{3!}{2!1!}$	8112
		1	1	1				52*42*12	$\frac{3!}{1!1!1!}$	157,248
			3					42*42*42	$\frac{3!}{3!}$	74,088
									Total	260,838

A critical point to note is that both the SRU and traditional unit cell representations have inherent limitations in terms of capturing long-range lattice-scale effects. Attempting to construct exhaustive unit-cell-based configurations over multiple repeated units (e.g., 9-unit cells) leads to an astronomical number of possibilities—on the order of 10^62^ for Si/Al = 5.00 using the unit cell as the representation, making direct simulation impractical. Our SRU method addresses this bottleneck by focusing on a manageable yet chemically relevant subset of configurations, leveraging topological symmetry and representative structural motifs. This approach allows for efficient exploration of configurational diversity without sacrificing the physical fidelity of adsorption predictions. The magnitudes of the reduced enumerations are reported in the Supporting Information.

As shown in Figure 3, we have applied the SRU representation to several zeolite frameworks, and the resulting SRU structures are smaller than the unit cell, offering a 2–4-fold reduction in the number of T atoms required in most zeolites. Figure 3 shows some examples of the SRU representation in contrast to the original lattice, while the unit cell is shown in gray. Note that the SRU structure has each T-node numbered. The numbering of the nodes in the SRU is based on the connectivity rules defined by the connectivity matrix. Figure 4 demonstrates an example of an SRU connectivity matrix for the SOD framework. The size of the matrix is governed by the total number of T-nodes in the SRU structure, and the connectivity matrix and the structure together make up the SRU representation. Following the connectivity rules, the entire lattice can be regenerated.

**Figure 3:**
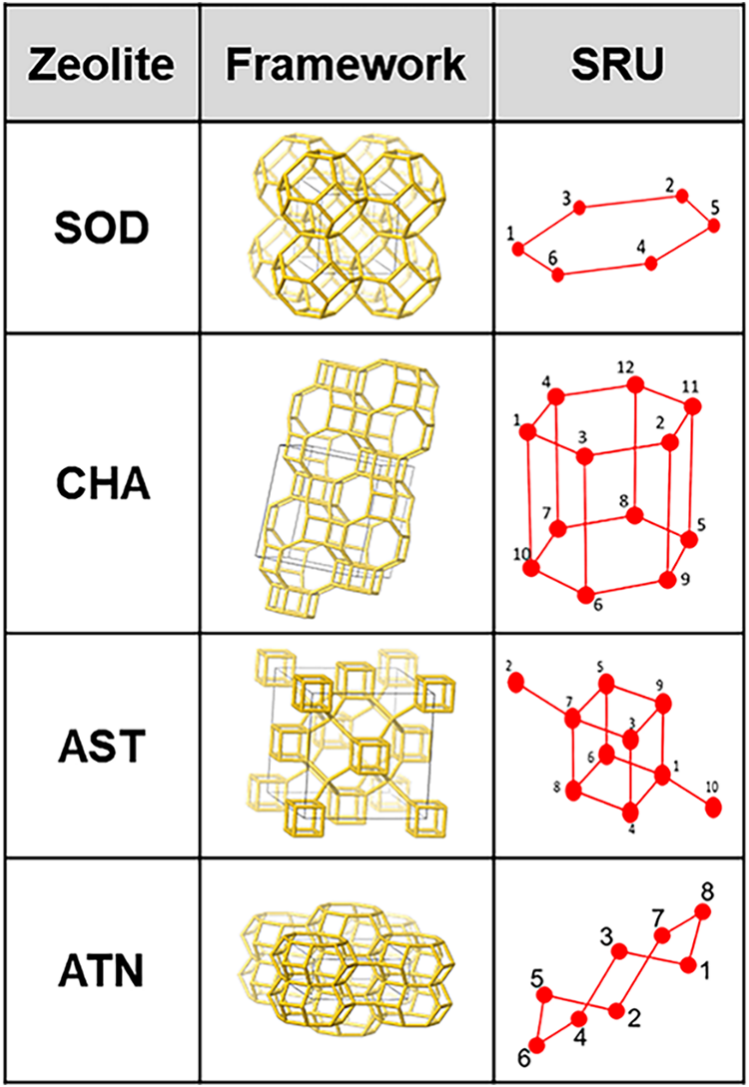
Examples of the SRU for a few zeolites. The unit cell has been highlighted in the framework, and the SRU structure is shown in red with the numbering based on the connectivity matrix.

**Figure 4:**
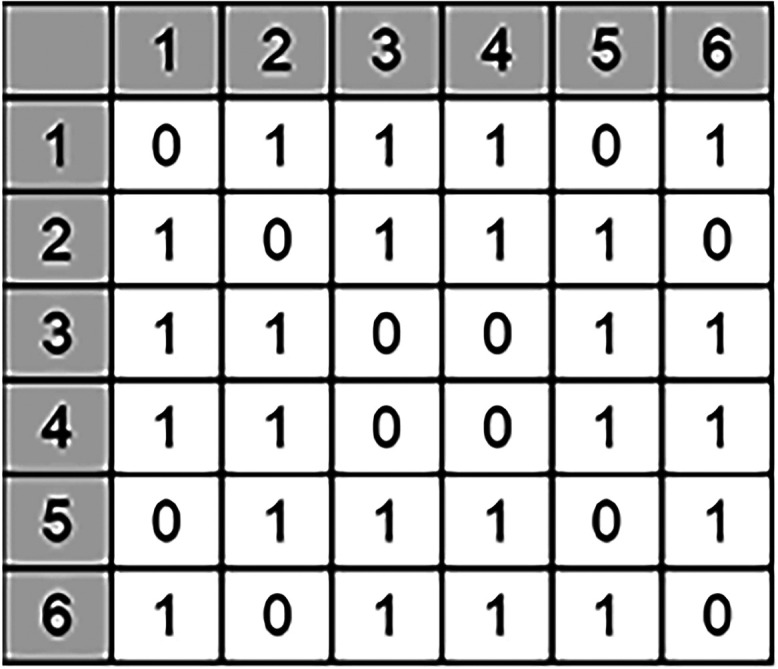
Connectivity matrix for SOD. The SRU structure has 6 nodes leading to a connectivity matrix of size 6 × 6. Note that the sum of all elements in a row and column is equal to 4 due to the tetrahedral nature of the T-nodes.

### 2.2 Rational Generation of Al-Substituted Zeolite Structures

With a clearer understanding of the SRU, we now focus on how this representation allows us to generate Al-substituted frameworks. The concept was briefly covered in our previous work, however, we only discussed the number of enumerations and did not establish the complete unit cell generation of these enumerated structures.^39^ To elaborate, we introduce the concept of a node index based on the neighboring 4 atoms in the lattice. We assign the node index to a value of 4 for a Si atom connected to all four Si atoms. The node index value is reduced by 2 for every neighbor that is substituted with Al until the node index goes down to −4. For a central Al atom connected to 4 Si atoms, we define the node index as −6. Note that in this entire definition of the node index, there is no value assigned for an Al–Al connectivity. This lies within the scope of our problem due to Lowenstein’s rule that forbids Al–O–Al linkage.

The node index is a theoretical concept that can be defined for each node in a structure. However, this concept can be mathematically programmed for matrices, which allows for the systematic enumeration of structures. In a mathematical framework, the node index can be defined as the sum of all of the row (or column since the matrix is symmetric) elements. With a pure Si framework, the node index for all of the nodes is 4 based on the tetrahedral nature. For example, we substitute site 1 with Al instead of Si. Mathematically, this means we multiply all elements in the row and column by −1 and update the diagonal element at (1,1) with a value of −2. Now when we recompute the node index for the entire matrix by summing the rows or columns, we get the same value as we would get from a visual inspection of the structure. Additional substitutions can then be performed on nodes only where the node index has a value of 4. The benefit of the representation and the node index lies in the fact that the computation can be mathematically programmed, thus making the process systematic.

To understand the framework in detail, let us first define the problem statement as given the SRU matrix for a zeolite, determine feasible Al substitution locations while obeying Lowenstein’s rule of no Al–O–Al linkage. We explain the solution procedure to obtain all feasible Al substitution locations via two functions described in Algorithms 1 and 2.

Algorithm 1 presents a function for adding a single Al atom to a given structure represented by a matrix *M* of dimension *m* × *m*. The algorithm returns a set of all feasible matrices that result from the substitution of a single Al atom. The function AddOneAl starts by initializing an empty set SSL that will store the feasible matrices. Then, the diagonal elements of *M* are extracted into vector *de*. The variable *s* is set to the highest index where *de* equals – 2, which corresponds to the last Al-substituted position site in the lattice. If no such site exists, *s* is set to 0, indicating that no Al substitution exists in the given matrix, and all positions should be considered for Al substitution.

Further, a loop over the indices *i* ranging from *s* to *m* is performed. At each iteration, a copy of *M* is created and stored in variable *G*. Then, the *i* ^*th*^ row and column of *G* are updated to represent the addition of the Al atom at empty site *i*. The update is only performed if the corresponding node index in the lattice is equal to 4, which ensures that the Al atom can be added without violating any constraints on the node index as defined previously. Finally, the updated matrix *G* is added to the set SSL . After the loop, the function returns the set SSL containing all feasible matrices resulting from the addition of the Al atom. Note that the function does not modify the input matrix *M*, as it creates copies of it for each update.
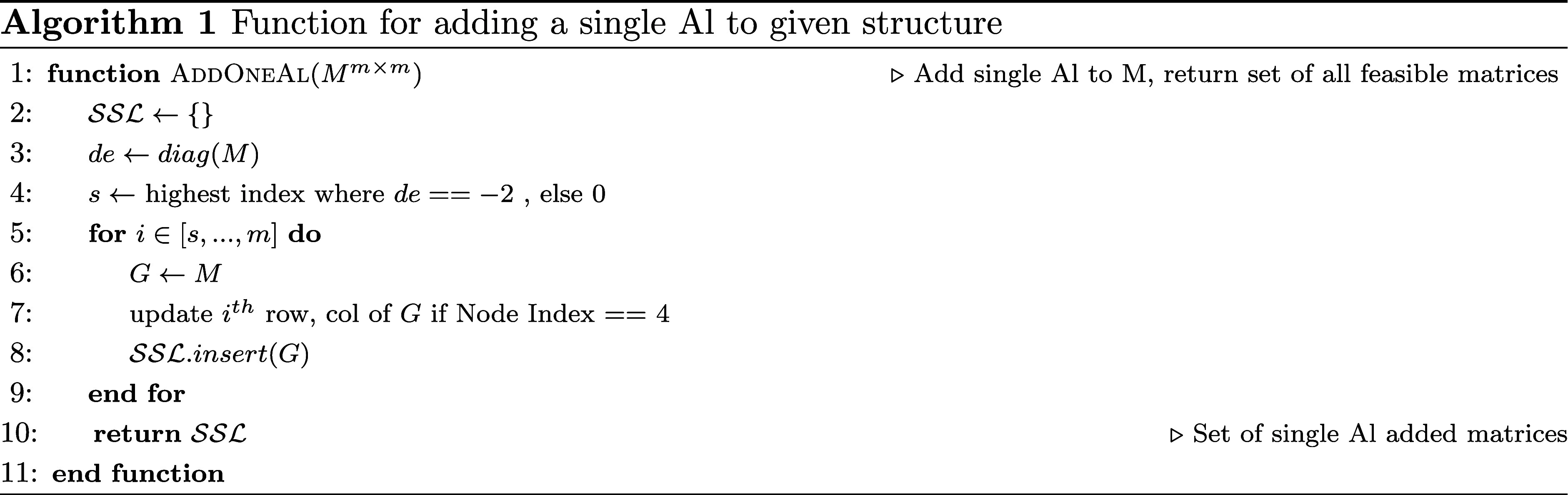


We now take a look at the most important function, where we determine the addition of all possible Al that is described in Algorithm 2. Algorithm 2 takes a connectivity matrix *C* and a positive integer *n* as input, and generates a set of matrices *A* with Al-substituted nodes, such that the number of Al substitutions is at most *n*.

The algorithm starts by initializing a copy of the input matrix *C* in *Z* as a temporary variable. The function GenMatrices is then called with *Z*, *n*, and an empty set L as input. If L is empty, the function checks if the number of nodes with value −2 (i.e., nodes that have not yet been substituted with Al) in *Z* is equal to *n*. If so, *Z* is added to the set A of matrices with Al-substituted nodes. Otherwise, the function AddOneAl is called to generate a set SSL of all possible matrices that can be obtained by adding a single Al to *Z*. For each matrix *I* in SSL , the function GenMatrices is called recursively with *I*, *n*, and SSL as input. This ensures that all possible combinations of Al substitutions are explored until the desired number of Al substitutions is reached.

If L is not empty, the function enters the recursive step. For each matrix *O* in L (which contains all possible matrices generated in previous recursive calls), the function AddOneAl is called to generate a set T of all possible matrices that can be obtained by adding a single Al to *O*. For each matrix, *T* in T , *T* is added to the set A of matrices with Al-substituted nodes. The function returns the set A of all matrices with Al-substituted nodes that are generated during the execution of the algorithm.
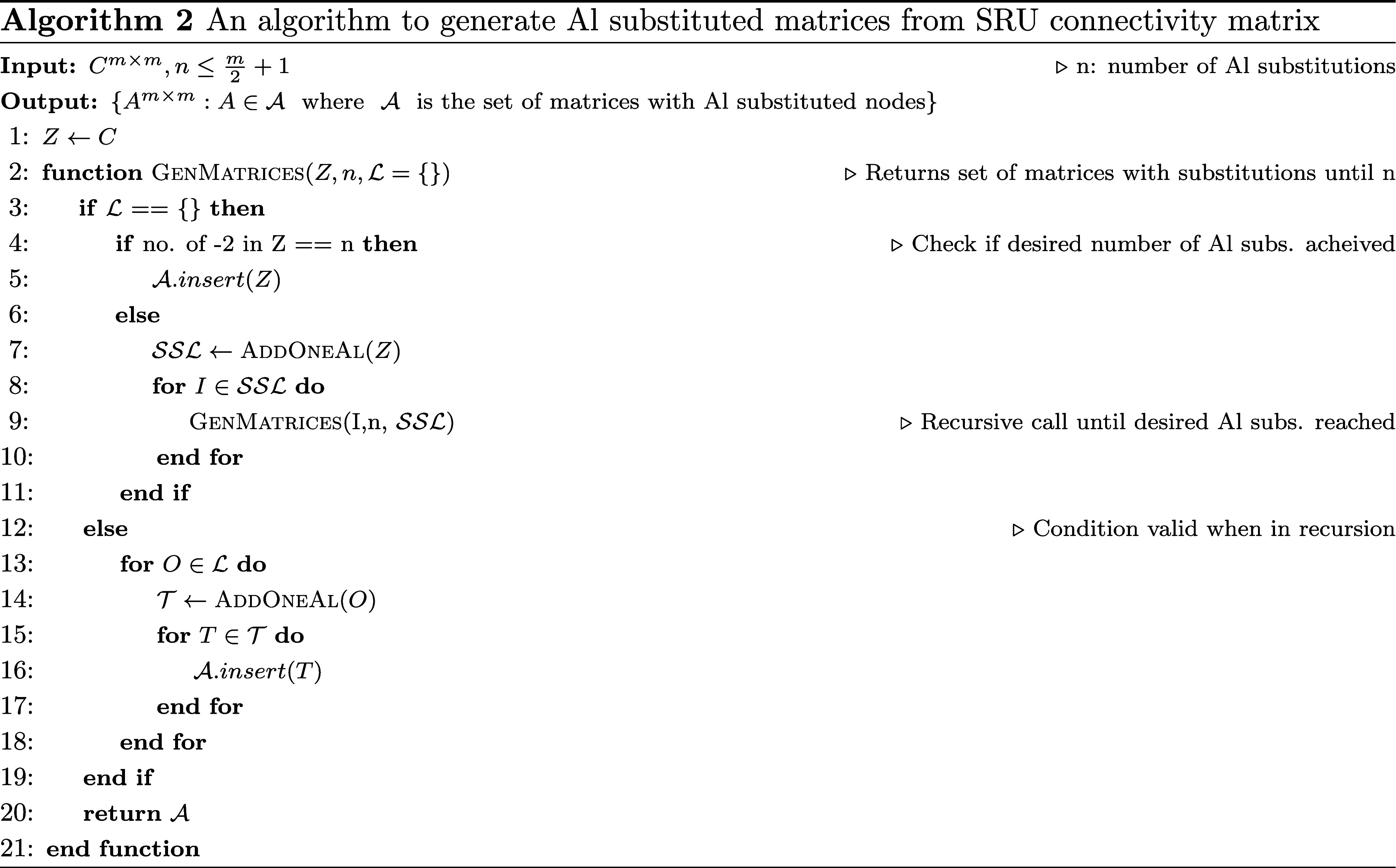


After generating the connectivity matrices, we then use the structure to generate lattice coordinates. The next step is to convert the lattice coordinates to CIF file structures for molecular simulations. Since most molecular simulation software uses the CIF format, we expand the lattice sufficiently large to ensure at least one unit cell is contained within and then identify the atoms within this volume using the original parallelepiped dimensions of the unit cell. We then imported these atoms as XYZ coordinates and converted the data to the CIF format. This process is critical since we want to exploit the periodic boundary conditions and mimic an infinitely repeating system to obtain accurate results. With the CIF structures generated, we can now perform molecular simulations to obtain adsorption isotherms for different Al substitutions. In the next section, we describe the molecular simulation methods that we use for our analysis.

As we present the different structures considered in this work, we show some examples of the unit cells that we generated using the SRU framework and the proposed methodology. Since the SRU of CHA has 12 T atoms, we generated structures from 1 substitution to 6, which led to Si/Al ratios ranging from 11.0 to 1.0, and these are shown in Figure 5. It is important to note that zeolites with Si/Al ratios of 1.0 have not been observed or synthesized, but considering these structures allows us to study the effects of Al substitutions computationally.

**Figure 5:**
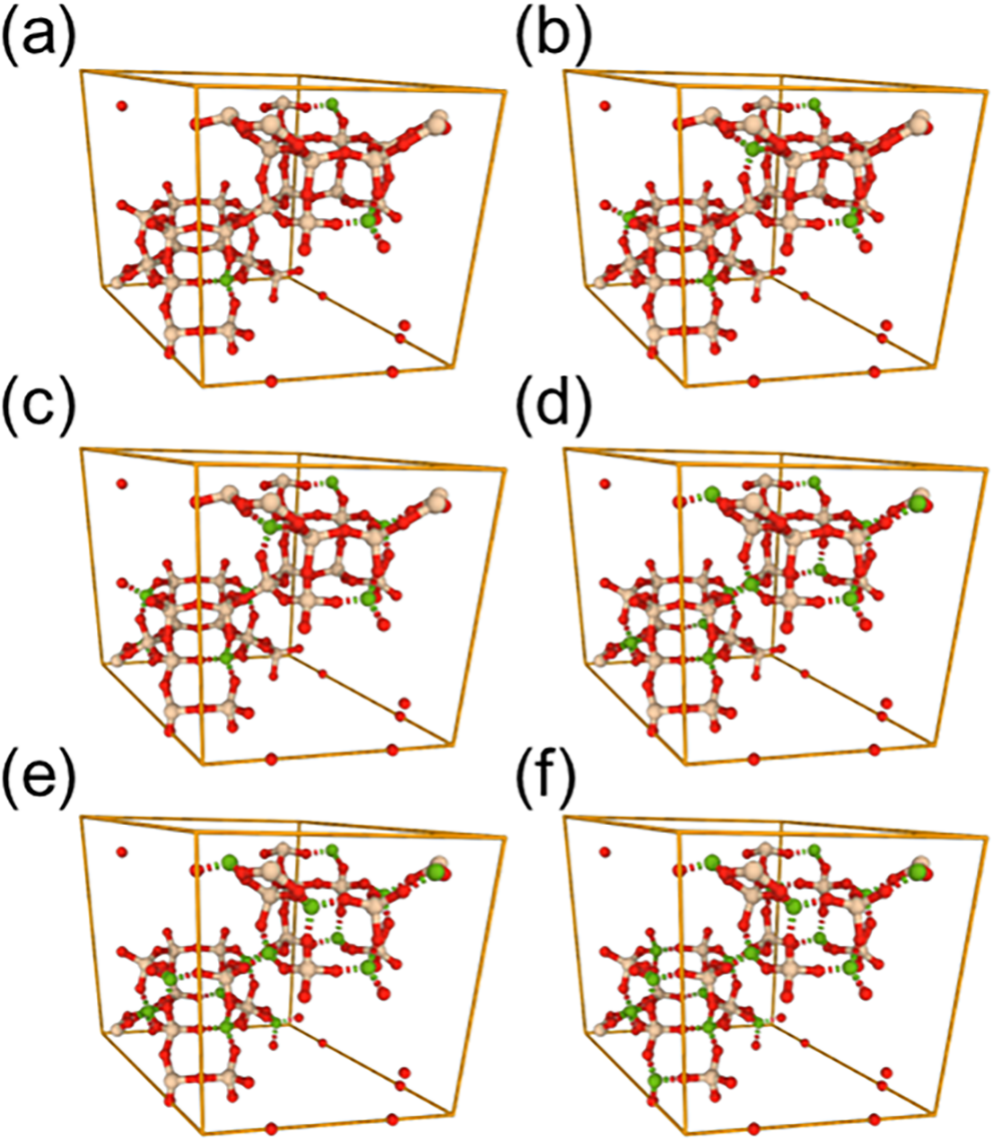
Examples of the unit cells of Al-substituted zeolite frameworks. Each cell has a different Si/Al ratio: (a) has a ratio of 11.0, (b) has 5.0, (c) has 3.0, (d) has 2.0, (e) has 1.4, and (f) has 1.0. Note the color scheme where Al is highlighted in green while Si and O are in ivory and red, respectively.

### 2.3 Molecular Simulation

To estimate the equilibrium gas adsorption capacity of the selected Al-substituted structures, we use the Monte Carlo simulation technique for sampling. Molecular simulations were performed using the multipurpose software RASPA 2,^41^ which is developed for microporous materials, to perform the Grand Canonical Monte Carlo (GCMC) simulations. GCMC simulations are performed for CO_2_ on Al-substituted zeolite frameworks at 298 K under varying pressures. The simulations follow the conventional techniques reported in the literature.^42,43^ The Lennard-Jones potential for the nonbonded interactions was taken from the Garcia Sanchez force field and is provided in the Supporting Information.^44^ Additional details of the atoms’ mass, charge, and radii are also provided in the SI. The CO_2_ molecules are based on the bond length and bond angle of 1.149 Å and 180°, respectively.

The simulations are initialized with 10,000 cycles and run for 25,000 production cycles. The thermodynamic properties are calculated every 1000 cycles. The simulation is performed in a 3 × 3 × 3 unit cell. The 3 × 3 × 3 simulation cells are generated using the VESTA software package, and the GCMC simulations are performed under periodic boundary conditions.^45^ Na ions are added to neutralize the system and allowed to float freely in the simulation box.^44^ The atom labels are retained, and the Al atoms connected to the oxygen atoms are renamed to identify themselves as separate atoms with interaction parameters obtained from the force field. For the ions, only translational and insertion probabilities are used with a value of 1 for both. For the adsorbate species, three moves are incorporated, which include translation, reinsertion, and swap, all with a probability of 1.0. The cutoff radius used in GCMC simulations was 12 Å.

After each simulation, the adsorbate’s absolute loading on the adsorbent framework is calculated and recorded. The simulations were conducted for a total of 150 frameworks for the range of Si:Al ratios generated. Each framework is simulated at 9 different pressure values: 1, 5, 10, 20, 30, 40, 50, 75, and 100 kPa at 298 K, resulting in a total of 1200 simulations. The chosen pressure values are selected based on their relevance in practical applications.^46^ The molecular simulations are performed at the High Performance Research Computing facility at Texas A&M University. The simulation time varies depending on the applied pressure. On average, each simulation takes about 60 h to complete on a computer with 4 cores and 32GB RAM.

We also compare the molecular simulation results with those observed in the literature. Due to their higher adsorption properties and stability, zeolites with some amount of Aluminum have been synthesized and studied in the literature.^9^ Impurities also skew the experimental results compared to the simulation results as shown in the study by Ghojavand et al.^46^ The closest experimental zeolite with minor impurities that studies adsorption of CO_2_ has been reported by Pourmahdi et al. where they report the adsorption of CO_2_ and CH_4_ on various CHA structures under different temperatures and pressures.^47^ The closest to pure-silica CHA is their reported zeolite S5 and the adsorption isotherm reported in Figure 6 of their work where we compare the adsorption at 298 K. Their experimental results on zeolite S5 are comparable to the simulation results reported in Figure 6 in this work with some margin for error for impurities and computational vs experimental errors.

**Figure 6:**
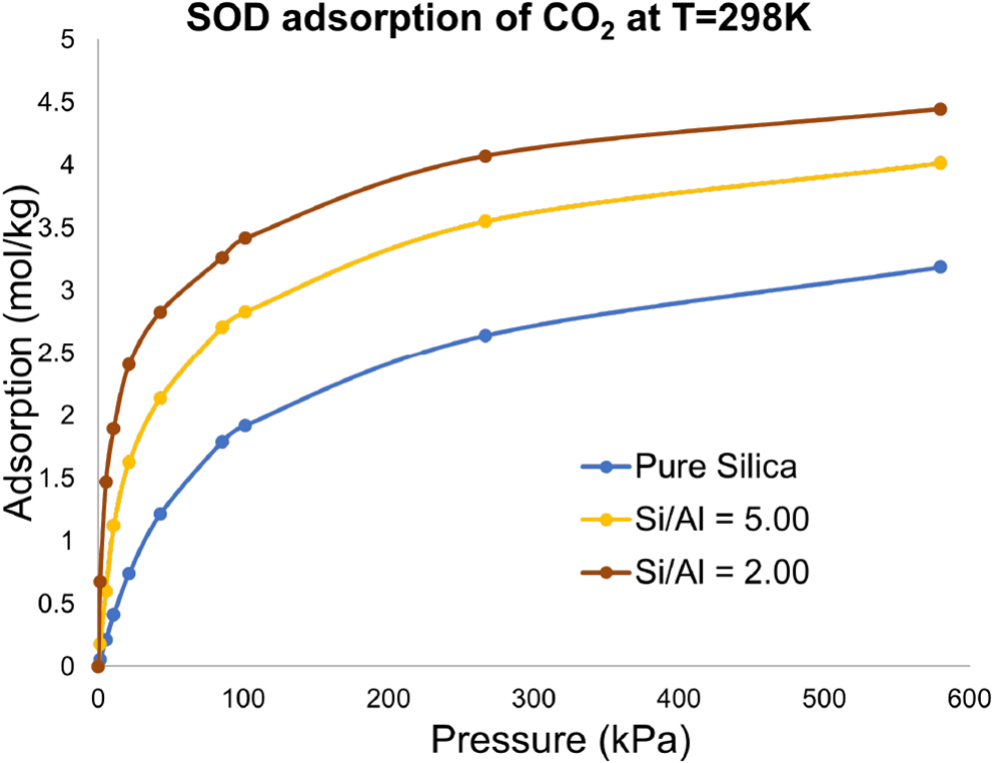
CO_2_ adsorption on the SOD framework with varying Si/Al ratios. The results are obtained using GCMC simulation.

### 2.4 Radial Distribution Function in Al-Substituted Zeolites

The effect of the proximity of Al atoms on the adsorption can be analyzed using the radial distribution function (RDF). The RDF, often denoted as *g*(*r*), is a fundamental tool in the study of materials that characterizes the distribution of particles around a tagged particle at a distance *r*. The RDF is a key indicator of the atomic structure of materials and varies significantly depending on the phase of the matter. It is therefore widely used for the characterization of solids, gases, and liquids. There are several possible RDFs, but we study only Al–Al RDFs in this work. In the case of materials with Al substitutions, such as zeolites, we focus on the Al atoms in the lattice and compute the RDF for these atoms. By analyzing the RDF, we can gain insight into the ordering and spatial arrangement of Al atoms in the material. The radial distribution function is defined as the ratio of ⟨ρ­(*r*)⟩, the average local number density of particles at a distance *r*, to the bulk density of particles, ρ, given by eq 1.

1 $$g\left(r\right)=\frac{\left\langle \rho \left(r\right)\right\rangle }{\rho }$$

The RDF is the computational method equivalent to the pair distribution function (PDF), which is obtained experimentally by using X-ray or neutron scattering. The two methods are the same; in fact, RDF is also called as the pair distribution function analogously. The PDF provides information on the positions of atoms in a crystal lattice and has soft peaks that correspond to high probabilities of atom presence. In contrast, the RDF has sharper peaks, since the positions of atoms are already known. The significance of the RDF in material characterization lies in its ability to provide information about the local environment of atoms and molecules in a material. This information is crucial for understanding the physical and chemical properties of materials such as their mechanical, electrical, and thermal properties. The RDF is therefore a valuable tool for materials science, chemistry, and condensed matter physics, and its continued development and application will be key in advancing our understanding of materials at the atomic scale.

Studying the RDF is similar to studying the PDF in that the focus is on the intensity and location of the peaks. The larger peaks in the RDF correspond to longer-range correlations in the lattice, while the first peak, typically the largest and most well-defined, corresponds to the nearest-neighbor distance between atoms. Subsequent peaks correspond to longer-range correlations between atoms, such as the second-nearest-neighbor distance, and so on. The smaller peaks observed in the RDF correspond to interatomic distances between the neighboring atoms in the lattice. These peaks are generally narrower and more well-defined than the broader peaks that correspond to longer-range correlations in the material. This is because the atomic positions in a crystal lattice are highly ordered and regular, resulting in sharper peaks in the RDF.

The algorithm for computing the RDF is described in Algorithms 3 and 4. The algorithm is given by using a parallel computation process. Algorithm 3 does all the data manipulation and then splits the data for each parallel process, which calls Algorithm 4. The final result is compiled and reported in Algorithm 3. In our case, the parameter *dr* is set to 0.25 since we have point locations of Al.
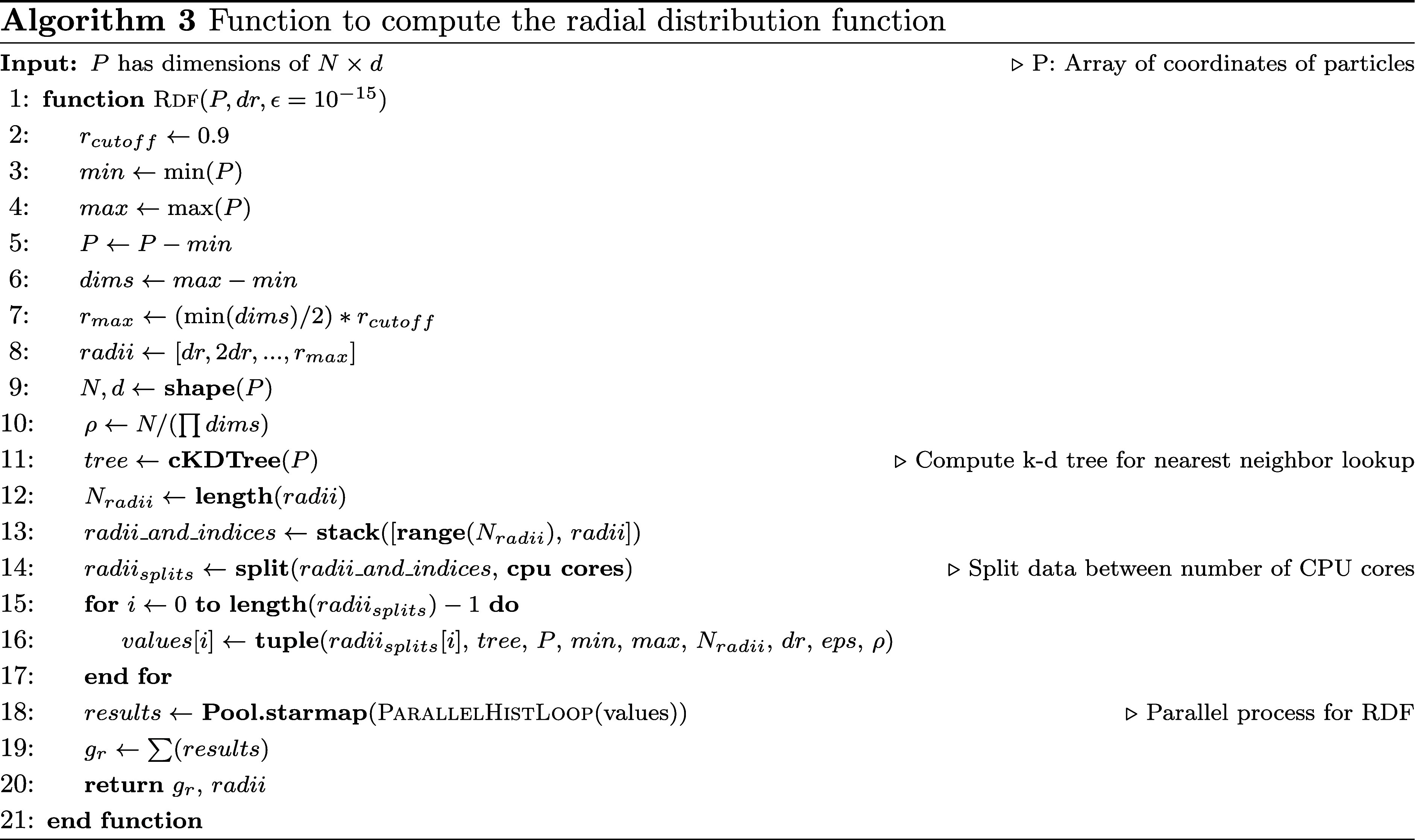
 
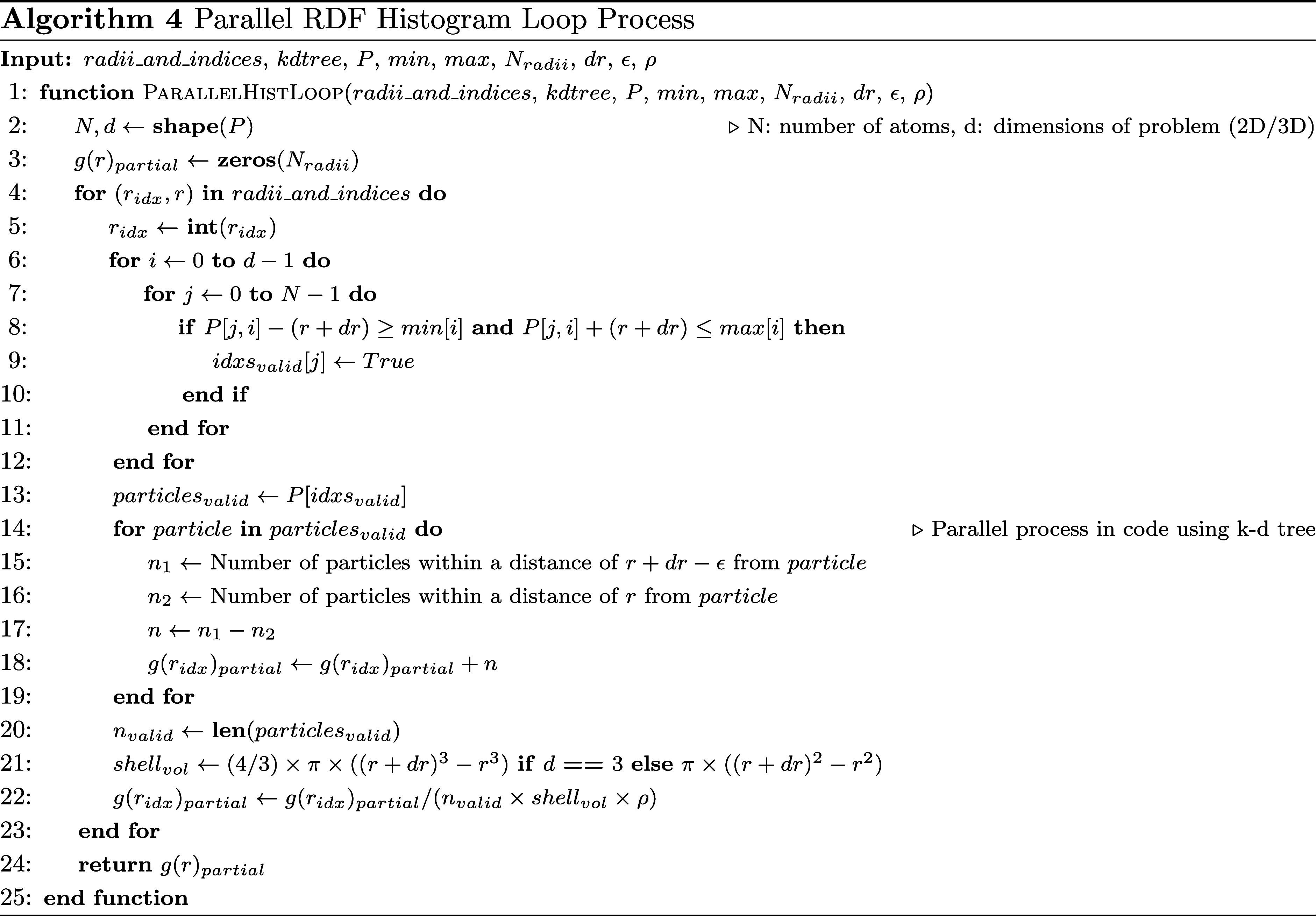


## 3 Results

### 3.1 Quantifying the Variation in CO_2_ Adsorption

We applied the proposed method to quantify the variation in CO_2_ adsorption in Al-Substituted SOD and CHA zeolites. First, we estimated the equilibrium adsorption of CO_2_ on SOD zeolite for different Al contents or Si/Al ratios. The Grand Canonical Monte Carlo (GCMC) based simulation results, as shown in Figure 6 for Si/Al ratios of 2 and 5 as well as for pure-silica zeolites, for which the ratio is very very large due to the absence of any Al atom. We see a significant increase in CO_2_ adsorption with increasing Al content in the SOD lattice, confirming the past observations^9,11^ that the presence of Al sites enhances CO_2_ adsorption. Among the designs, we achieve the highest adsorption for a Si/Al ratio of 2. We could not decrease the ratio (or increase the Al content) further. For example, a Si/Al ratio of one for SOD zeolite is infeasible according to the Lowenstein rule. The Si/Al ratio of 2 is the lowest that can be achieved with the SRU representation for SOD. A unit cell level representation would give us a ratio of 1.4 but will generate 792 possibilities, which can be reduced by eliminating repeating structures, but the number will still be high.

We perform a similar study of CO_2_ adsorption on the CHA zeolite framework for different Al substitutions. The variations in CO_2_ adsorption on the CHA zeolite framework for all selected Si/Al ratios are shown in Figure 7. For the same Si/Al ratio, the width of the band represents the variations in adsorption amount due to different enumerations of Al positions in the lattice. For a large Si/Al ratio (e.g., Si/Al ratio equal to 11), we have a narrow width, indicating small variations in adsorption. This is because the distributions of Al sites are sparse and, in most cases, they are located far from each other, thereby nullifying the effect of the geometric locations of Al sites within the zeolite framework. Most of the T-sites are Si atoms, and there is little variation in the overall composition of the framework in terms of Al density. Similarly, we observe little variation in CO_2_ adsorption when the Si/Al ratio is small (e.g., Si/Al = 1.0) due to the lack of compositional variation in terms of Si as most T-sites in this case consist of Al atoms.

**Figure 7:**
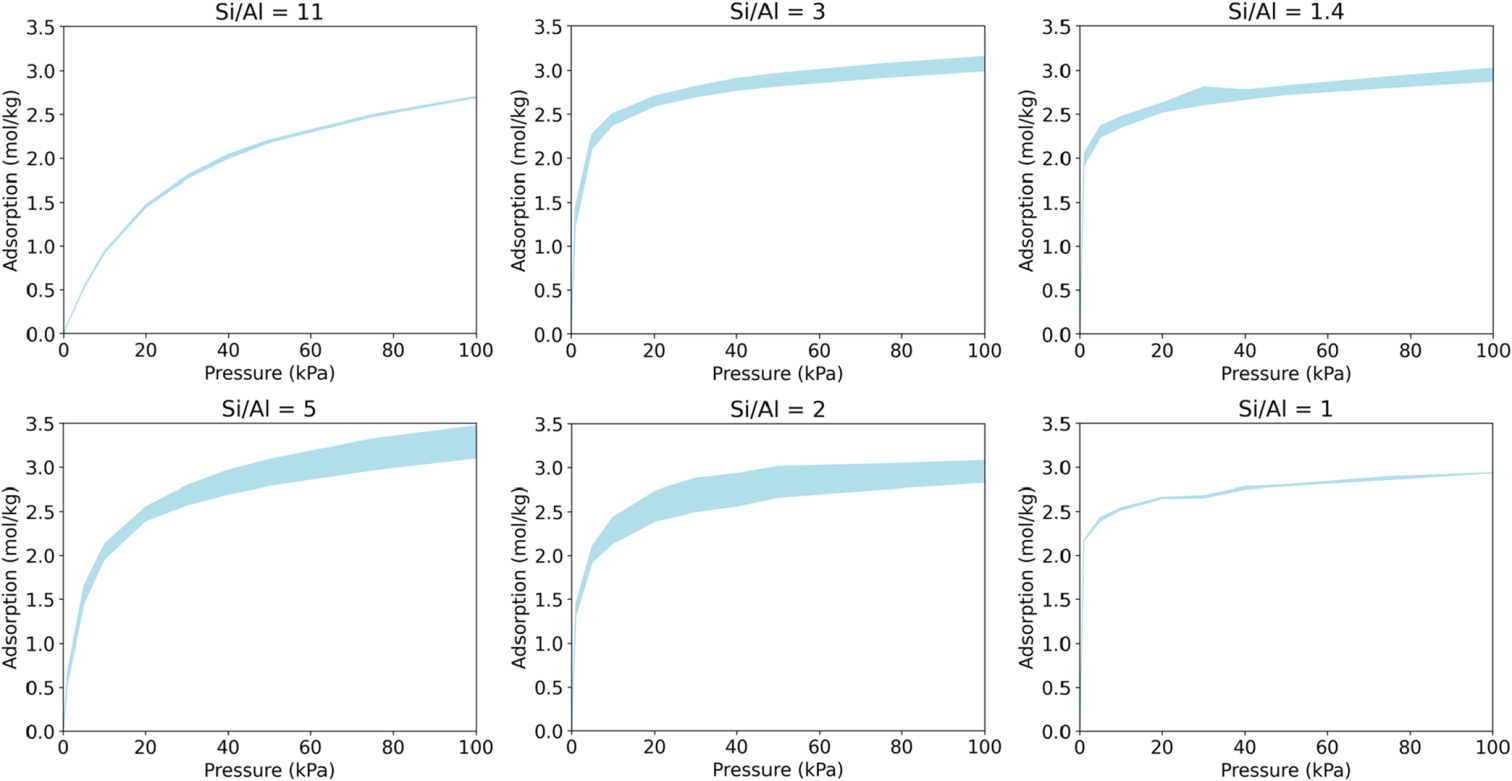
Variability in CO_2_ adsorption on the CHA framework with different Si/Al ratios.

A different result is obtained for moderate Si/Al ratios. The variations in CO_2_ adsorption are most prominent at a moderate Si/Al ratio. For example, we observe large variations in adsorption amount for Si/Al ratios of 2 and 5. We hypothesize that this can be due to the larger variations in Al density within the zeolite framework. For the same Si/Al ratio, the number of Al atoms per unit cell would be the same, but the locations of the Al sites within the unit cell would be different for different Al distributions. Due to this, denser clusters of Al sites in specific locations may considerably enhance the adsorption of CO_2_ compared to structures with more evenly distributed Al sites. This disparity in adsorption affinity is prominent when different clusters of T atoms are present that gives rise to nonuniform distribution of Al sites. Therefore, we predict that the variability in the amount of CO_2_ adsorption would be large for moderate Si/Al ratios. We also observe that the variations are large at higher adsorption pressures. This is expected as higher system pressure allows higher adsorption for disparately located denser clusters of Al sites, thereby increasing the differences in local adsorption within the unit cells.

We also observe that the CO_2_ adsorption does not always increase with the number of Al sites, and there exists an inflection point beyond which additional Al substitution leads to a decrease in adsorption. For CHA, the CO_2_ adsorption is maximum when Si/Al is 5.0 and drops on further Al addition. This result is consistent with previous studies in GIS-type frameworks.^9,10^ The adsorption capacity for different Al substitutions per SRU cell (12-sized SRU for CHA) at 298 K and 100 kPa is shown in Figure 8. The maximum adsorption is observed at a Si/Al ratio of 5.0. Note that for this fixed ratio, there is a significant variation observed as well, and we aim to quantify this variation and study the cause for it.

**Figure 8:**
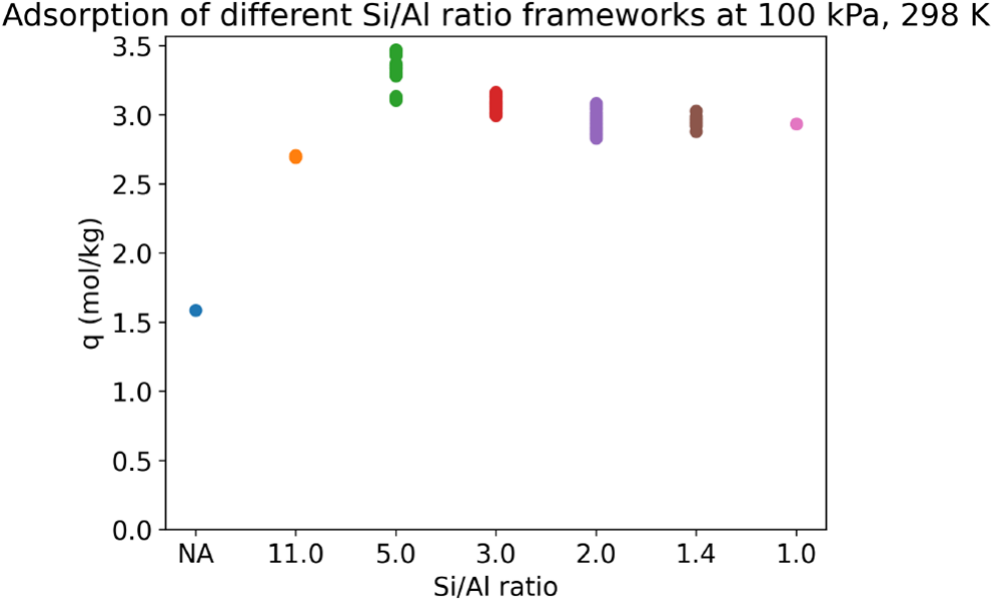
CO_2_ adsorption isotherm for CHA frameworks with different Si/Al ratios at 100 kPa, 298 K.

To understand the physicochemical factors behind the optimal amount of Al that results in the highest adsorption, we need to examine the lattice structure. In a pure-silica lattice, the structure contains crevices for adsorption formed by the tetrahedral arrangement of Si and four oxygen atoms. When some of the Si atoms are replaced with Al, additional cations must be incorporated into the lattice to maintain charge balance, and these cations occupy certain lattice sites. The introduction of Al creates a charge field, enhancing the adsorption. However, with further Al substitutions, the cations begin to occupy the CO_2_ adsorption sites, leading to a decrease in adsorption. Therefore, there is an optimal amount of Al that maximizes adsorption before the charge-balancing cations occupy the adsorption sites.

We further investigated the effect of positions of Al on adsorption for the fixed Si/Al ratio of 5.0. There are 42 Al-substituted configurations of CHA zeolite frameworks with this ratio that are generated using the SRU approach. The variation observed in this case is significant, ranging from 3.10 to 3.47 mol/kg at 100 kPa. This accounts for 12% variation in the adsorption. Figure 9 shows the variation in more detail, and we can clearly see individual isotherms, some of which have higher adsorption than others. Few select unit cells of CHA zeolite with a Si/Al ratio of 5.0 are shown in Figure 10. Each of the structures shown has exactly 6 Al atoms in the unit cell. However, due to differences in Al locations, they lead to different amounts of adsorption of CO_2_.

**Figure 9:**
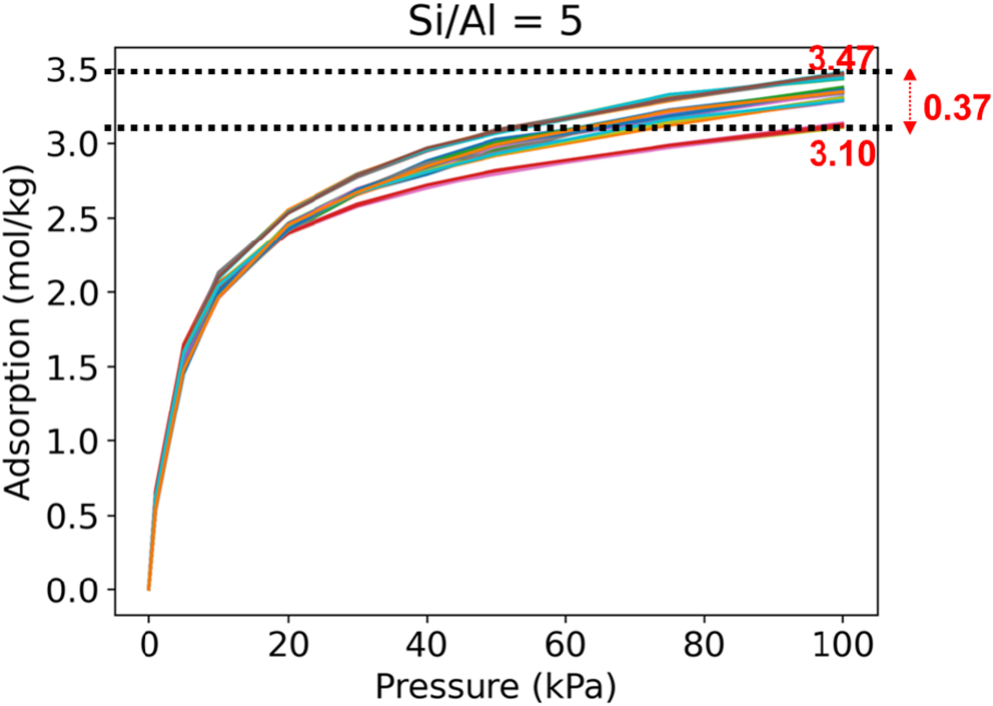
Variation observed in CO_2_ adsorption on CHA frameworks with Si/Al = 5.0 and different Al substitution locations.

**Figure 10:**
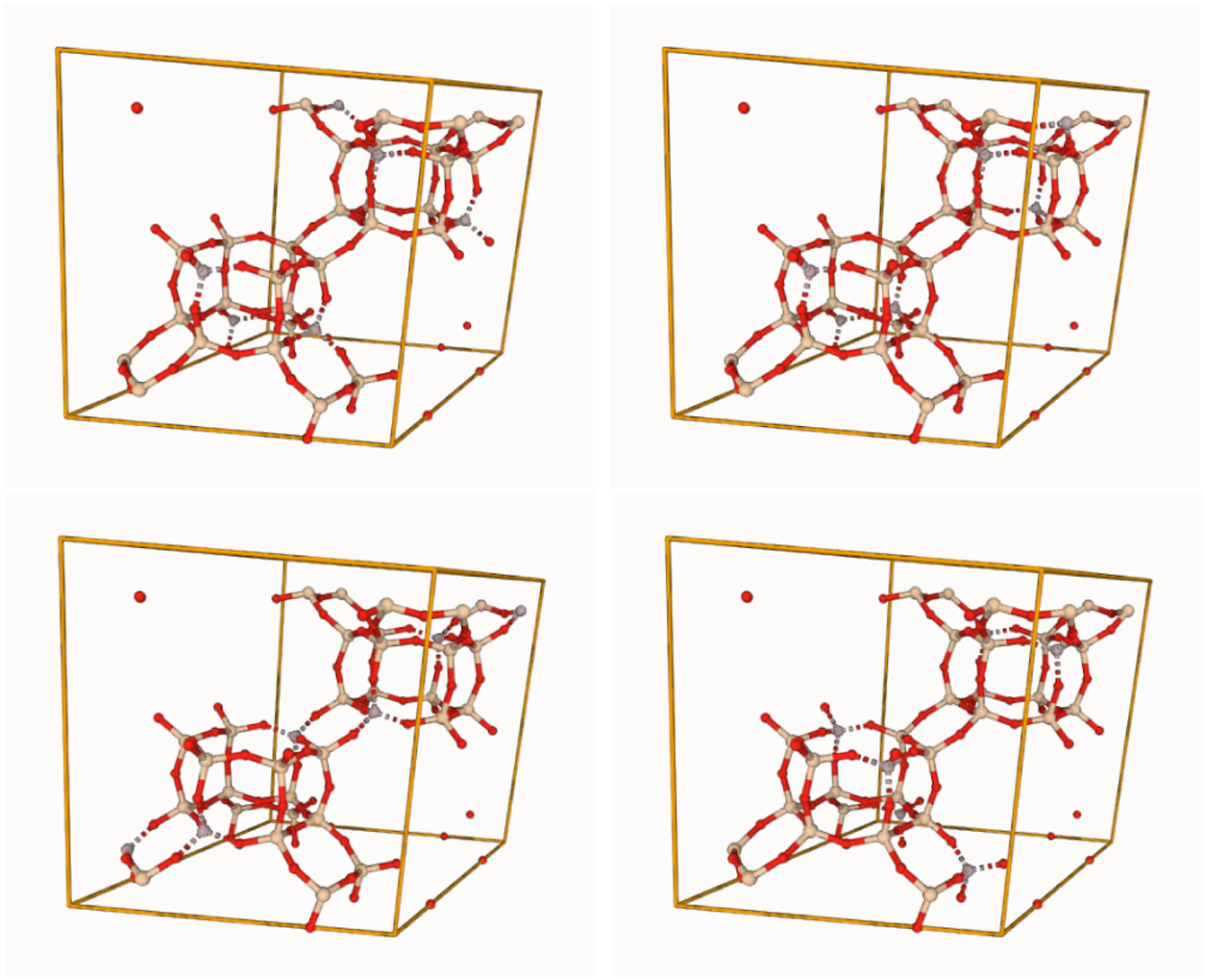
Examples of different Al-substituted CHA framework configurations with Si/Al ratio of 5.0. The colors for Al, Si, and O are gray, ivory, and red, respectively. The bonds are depicted via dashed lines.

While current CO_2_ capture costs are estimated between USD 15 and 120 ton^–1^ CO_2_,^48^ a 12% improved adsorption capacity will have a significant reduction in the costs. The implications on the process operability are even more critical as has been shown in the literature.^49^ A process designed to operate with a certain expected material performance in terms of purity and recovery will need significant modifications to operate at materials with properties differing by 12%. This is critical for not only simulation-based study but also for optimization with processes that have narrow operability domain. Even though many hypothetical zeolites and crystalline materials have been proposed in the literature previously, the high cost of experimental synthesis has hindered active research in this domain. Though the example selected in this study has focused on CO_2_, zeolites are highly effective in selective adsorption; thus, targeted adsorption of contaminants can also benefit from this approach. The low-density nature of zeolites results in a lighter weight in the overall setup, facilitating ease of handling and potentially allowing higher additive loading.

We use the RDF to study how the locations of Al affect adsorption. For the CHA framework with Si/Al = 5.0, we have 42 different configurations of Al distributions, based on the SRU representation. Of these, some are repeated due to the orientation of the 3-dimensional unit cell during the systematic enumeration. By observing the differences in the RDF profiles of these structures, we classify them into six groups. These six different groups of RDF profiles are shown in Figure 11 along with the adsorption isotherms of the corresponding framework configurations. In all the RDFs, the highest peak is observed at 9.25 Å, which indicates the most frequently observed distances between two Al atoms in the lattice. Note the dimensions of the unit cell of CHA are *a* = 13.675 Å, *b* = 13.675 Å, *c* = 14.767 Å, α = 90°, β = 90°, γ = 120°, so the peak signifies the Al position due to repetition of the SRU. The peak at 12.5 Å also is observed in all RDF profiles. Since the unit cell has 6 Al substitutions (Si/Al = 5.0), we are interested in the five tallest peaks since we are looking at other Al from one of the Al. Looking at the RDF of groups 4 and 5 (Figure 11d,e), we observe that all of the peaks are observed at the same values. However, due to the numerical precision floating decimals, there is some nonzero value at 5.75 Å. Thus, by visual inspection, we can still classify these groups together. Looking at the rest of the groups, we see significant differences in the locations of the peaks. Groups 4, 5, and 6 (Figure 11d–f) all have a peak at 4.25 Å. Group 6 (Figure 11f) does not have the peak at 5.5 Å. Groups 1, 2, and 3 (Figure 11a–c) all have their first peaks at much further distances than 5 Å.

**Figure 11:**
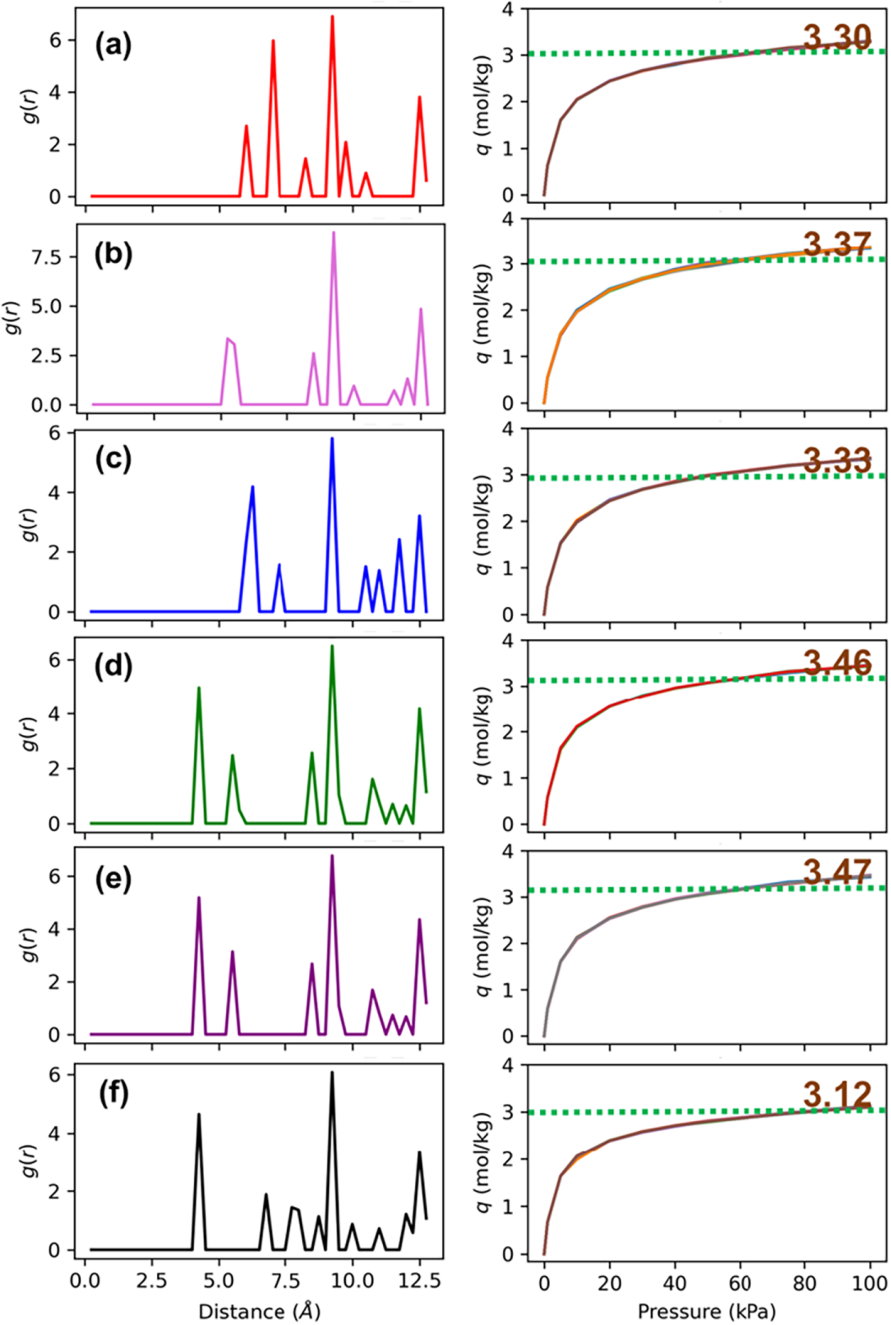
Radial distribution function *g*(*r*) for: (a) group 1, (b) group 2, (c) group 3, (d) group 4, (e) group 5, and (f) group 6 shown on the left side and the corresponding adsorption isotherms shown for each group on the right side. All RDFs correspond to the same material class, CHA, with a Si/Al ratio of 5.

The characterization of the positions of Al in the lattice is important to observe what factors lead to higher adsorption and the contrasting factors that lead to lower adsorption. With that goal, we also plotted adsorption isotherms from the GCMC data for each of these groups individually. These are shown on the right column in Figure 11. There is not much variation in the adsorption isotherms for the Al-substituted configurations with similar RDF. This justifies our classification of Al-substituted configurations in groups based on RDF. The acceptable variation for structures from the same group is 2% due to the stochastic nature of molecular simulations. Furthermore, groups 4 and 5 have very similar adsorption isotherms. This is expected due to the similarity in the RDFs for these groups. These groups (4 and 5) also show the highest saturation capacity or maximum equilibrium adsorption (3.46 and 3.47 mol/kg, respectively, at 100 kPa). Both the extreme adsorption isotherms, i.e., the least and highest, are observed when the second peak in the RDF is at 4.25 Å (groups 4, 5, and 6). On further inspection, we see that the highest adsorption is observed when a peak is present at 5.5 Å. For groups 1, 2, and 3, since the adsorption isotherms are not extremes, we can comment on the features that stand out for all of them. Especially in group 2, which has the next best adsorption, shows a peak at 5.5 Å. Thus, we can infer that a peak at 5.5 Å increases adsorption and, when combined with a peak at 4.25 Å, the adsorption observed is highest (3.47 mol/kg).

### 3.2 High-Throughput Screening of Al-Substituted Zeolite Configurations

The immediate next question is how do we use these insights to screen and select the structures with high adsorption from the large search space of over 2 million structures that exist for the same Si/Al ratio of 5.00. Note that insights generated from this analysis will also help experimental synthesis since only a few researchers are capable of synthesizing zeolites with Al located at selected sites.^50^ With these insights, we screen among the 2 million structures and verify the validity of the filters that we identify, thus proposing novel material designs with the desired properties. A quick preview of the screening is shown in Figure 12. The first screening criterion is the Löwenstein’s rule to the filter structures in which the Al–O–Al bond exists. This filter reduces the solution subset space to just 259,822 structures. Next, we introduce the criteria for the presence of a peak at 4.75 and 5.5 Å sequentially. Note that instead of hard constraints on the peaks, we allow for some flexibility by the constraints that a peak should be less than 5 Å and another peak between 5 and 5.75 Å. The first filter reduces the search space by 13% to 226,469, and the second peak filter further reduces the subset size by 11% to 201,622.

**Figure 12:**
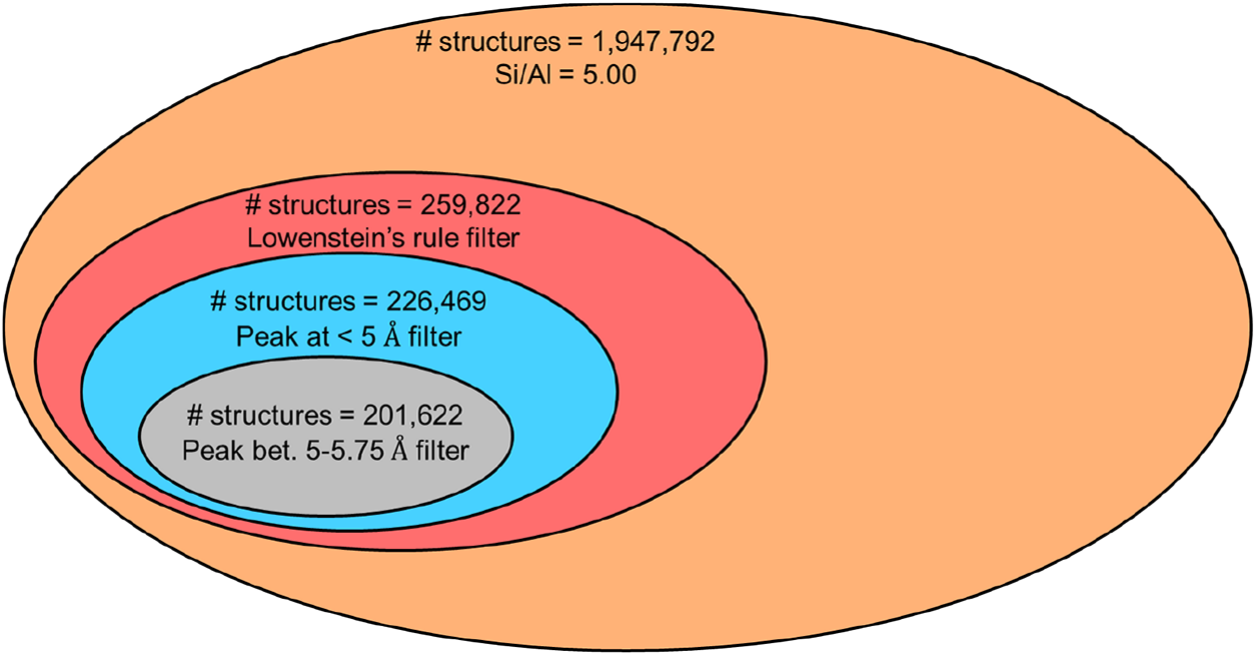
Subsets based on filters applied to 2 million ways of generating Al-substituted structures.

While the peaks at 4.75 and 5.5 Å have been observed for the CHA framework, the values at which peaks occur may vary for other zeolite frameworks. However, the methodology shown in this work can be applied to the zeolite framework of interest to identify the optimal Si/Al ratio, followed by the rest of the approach to identify the peaks. The contribution of this work highlights the specific case of CHA but expands beyond the single example considered. Using the SRU structure, one can reduce the potential structures and, following this analysis, can identify zeolites with desired properties.

To further screen from the selected subset based on the RDF profiles, we compare similar RDF profiles and observe the adsorption capacity of the corresponding structures. To compare RDF profiles, we use the cosine similarity and select the top 20 structures that have RDF similar to groups 4 and 5. The cosine similarity between two vectors, in this case, the RDF profiles **A** and **B** can be computed using eq 2.

2 $$\mathrm{cosine}\;\mathrm{similarity}\;\left(A,B\right)=\frac{A\cdot B}{∥A∥∥B∥}$$

where **A**·**B** represents the dot product of vectors **A** and **B**, and ∥**A**∥ and ∥**B**∥ represent their respective Euclidean norms. With the top 20 structures, we run molecular simulations for all of these, of which the minimum adsorption is 3.17 mol/kg and four of the top 20 structures have adsorption between 3.45 and 3.48. Note that the cosine similarity is just one of many metrics considered for vector comparisons. Further investigation into the top 20 structures shows that when we have similar RDF profiles (additional similarity check based on visual inspection for the intensity of the peaks), the adsorption is very high. The top four structures with high adsorption and similar RDF profiles are shown in Figure 13. The benefit of obtaining multiple structures that may lead to higher adsorption is of significant importance for experimental synthesis which allows for some flexibility in synthesizing among multiple structures. The RDF provides a unique mapping for the structure–property relationship.

**Figure 13:**
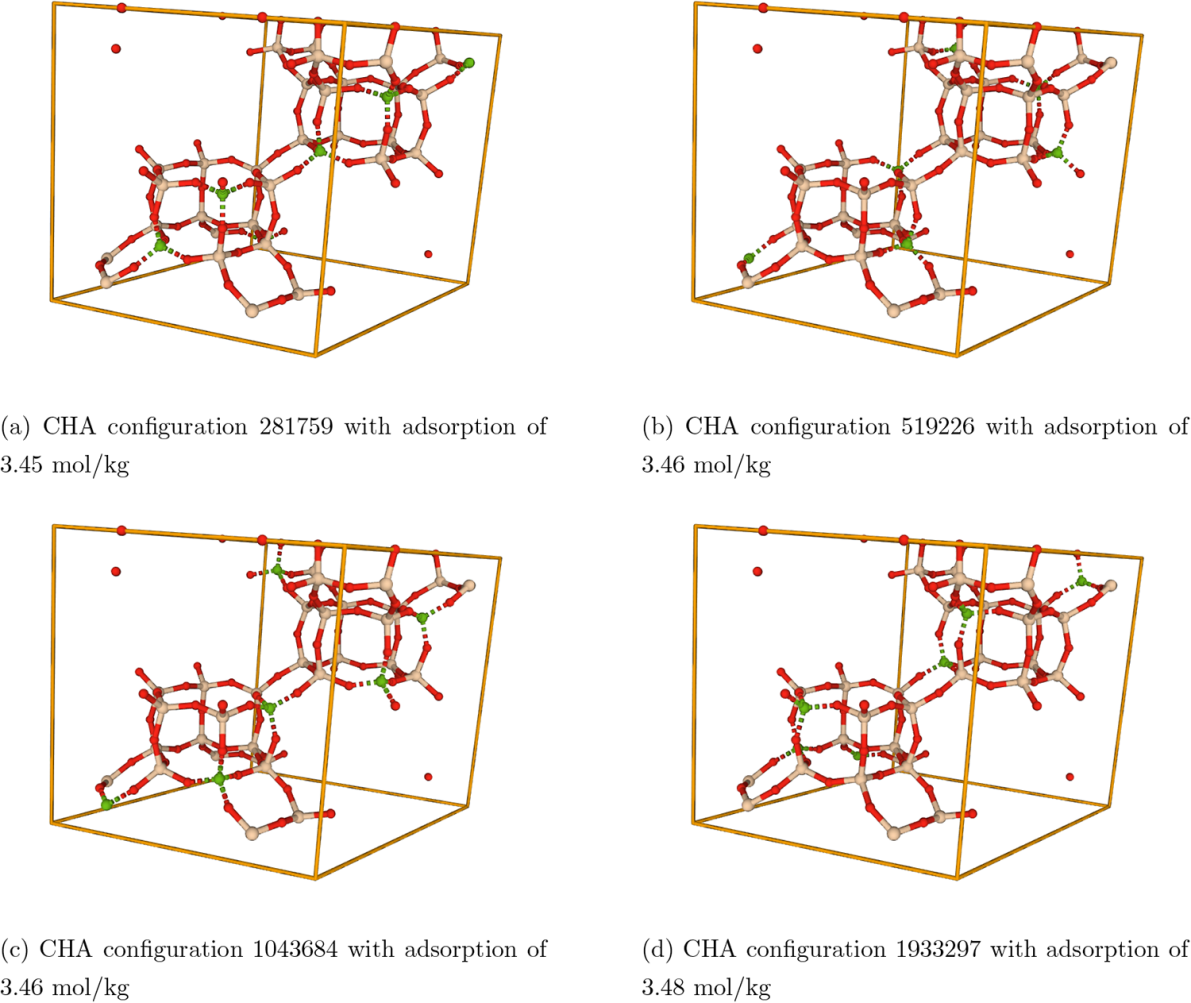
Top four identified configurations of the Al-substituted CHA framework with high *CO*_2_ adsorption at 298 K and 1 bar discovered via the proposed RDF-based high-throughput screening approach. All RDFs correspond to the same material class, CHA, with a Si/Al ratio of 5.

The physical inferences of the peaks at 4.75 and 5.5 Å are important to understand the underlying mechanisms of adsorption. On taking a closer look at the RDF profiles for SRU groups, we observe a high-intensity peak at 9 Å. The repetition distance of the SRU structure is responsible for this peak. Specifically, the location of a specific node between two SRUs leads to a high-intensity peak. Given the two peaks closer to 5 Å and another two peaks around 8.5 Å, the physical interpretation is that Al are locally close with some distance between them. This can be interpreted as some uniform distribution of Al in the lattice. To verify how the Al density affects adsorption, we also look at two structures with high local Al density. The structures are shown in Figure 14. The Al atoms are highlighted in green for the sake of contrast. Molecular simulation results for these structures validate our hypothesis of the uniformity of the distribution of Al. These structures show adsorptions of 3.15 and 2.7 mol/kg with lower adsorption observed for the densely packed Al. However, no comment can be made on the quantification of the uniformity, and this requires large data of molecular simulations.

**Figure 14:**
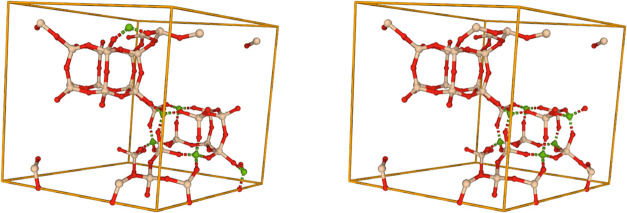
CHA structures with Si/Al = 5.0 but local high-density Al-substituted frameworks. Al atoms are shown in green.

## 4 Conclusions

We provide an efficient method for quantifying the variability in equilibrium gas adsorption on aluminosilicate zeolites due to the distribution of aluminum atoms within the framework. Such variability introduces considerable uncertainty in gas separation and storage applications, emphasizing the need for a systematic approach to address these challenges. We introduced a computational framework, based on a new representation of zeolite frameworks using single repeating units (SRUs), to perform the efficient and selective enumeration of unique Al-substituted configurations. This computational approach avoids exhaustive searches while providing insights into the equilibrium gas adsorption variability. Applying this methodology to CO_2_ adsorption on SOD and CHA zeolites, we observed considerable variation in CO_2_ adsorption based on Al atom locations, with the variability being most pronounced at moderate Si/Al ratios. At very high or low Si/Al ratios, the adsorption variability diminished. Our findings also reveal a nonmonotonic relationship between the number of Al sites and CO_2_ adsorption. Beyond a certain point, additional Al substitution reduces the adsorption, highlighting the existence of an optimal Si/Al ratio for maximizing the CO_2_ uptake. Through systematic analysis, we can identify this optimal ratio and the corresponding Al site configurations that maximize adsorption performance.

As with any representation, the SRU has limitations in that not all Si/Al ratios can be enumerated with a single unit. In those cases, multiple SRUs can be combined to make a larger unit to represent these cases. With the enumerated structures, we performed GCMC simulations to obtain the adsorption isotherms for the same and study the variation that we initially hypothesized. Experimental data are not yet available for these cases, and thus, the GCMC parameters, specifically the interaction parameters, may require further tuning to match with experimental data. However, a similar trend in variation in adsorption is expected to be observed in experimental results.

Our investigation further used radial distribution functions (RDFs) to pinpoint specific Al site arrangements that enhance the CO_2_ adsorption. These results underscore the utility of the SRU-based selective enumeration combined with RDF-based screening as a robust tool for rationally designing zeolites with tailored Al distributions. This approach paves the way for developing Al-substituted zeolite materials optimized for gas separation and storage applications. RDFs are found to be effective in describing the structural effects on the variation in gas adsorption. For example, upon studying the RDF of CHA zeolite, we find that the distinguishing factor for high adsorption is the first two peaks being observed closely at 4.25 and 5.5 Å. Due to the unique mapping of the RDF, this representation can be used for modeling the structure–property relationship. The key contribution of this study is the methodology that allows identifying the optimal Si/Al ratio and the aluminum RDF distribution, which is demonstrated via the case study of CHA. Further, if zeolite synthesis can be done with targeted Al substitution sites, then we can extend the current approach to discover new zeolite configurations with optimal desired properties.

## Supplementary Material



## Notes

Number of possible Al substitutions in various zeolite frameworks at given Si/Al ratios, force field and interaction parameters for the GCMC simulations for CHA and SOD, and atom descriptions of the atoms present in the lattice for both CHA and SOD (PDF)
