# Supplementary material for: Quantifying Gas Adsorption Variability and Optimal Si/Al Ratio for Rational Design of Aluminum-Substituted Zeolite Frameworks
Source: Langmuir. 2025 Jun 11;41(24):15255–69. doi: 10.1021/acs.langmuir.5c00276 (PMC12199476; doi:10.1021/acs.langmuir.5c00276)
Supplement: Supplementary file 1 [file la5c00276_si_001.pdf]

Quantifying Gas Adsorption Variability and Optimal Si/Al Ratio for Rational Design of Aluminum Substituted Zeolite Frameworks

Akhilesh Gandhi[a], **Silabrata Pahari**[a], **Joseph Sang-II Kwon**[a], **M. M. Faruque Hasan**[a,b,*]
[a]Artie McFerrin Department of Chemical Engineering, Texas A&M University, College Station, TX 77843-3122, USA.
[b]Texas A&M Energy Institute, Texas A&M University, College Station, TX 77843, USA.

Supporting Information

List of Tables

S1 Number of possible Al substitutions in various zeolite frameworks at given Si/Al ratios. 1
S2 Force field interaction parameters for the Garcia-Perez force field[1,2] 3
S3 Atom descriptions for GCMC simulations in SOD framework 4
S4 The non-bonded interaction parameters for GCMC force field parameters 4
S5 The list of atoms in the lattice. Data for their mass, charge, and radii are given. . . . 5

1 Benefit of SRU representation in enumeration of feasible structures

The benefits of the SRU representation in terms of reduced representation and Al-substitution are highlighted in our previous work.[3,4] In addition to the previous reported numbers, we also report the enumerations for other zeolites with different Si/Al ratios in Table S1. As demonstrated in the table, we report 2 Si/Al ratios for three zeolites, CHA, AST, and APD. The reduction provided by the SRU representation is significant because of the criteria of the Lowenstein's rule forbidding Al-Al linkage. The reduced representation of the SRU, though does not contribute to the reduction of possibilities, helps in molecular simulations where we assume uniform Al distribution.

Table S1: Number of possible Al substitutions in various zeolite frameworks at given Si/Al ratios.

Zeolite	Si/Al Ratio	# of Possibilities with Unit Cell	# of Possibilities with SRU
CHA	5.00	1,947,792	260,838
CHA	2.00	1,251,677,700	132,204
AST	4.00	76,904,685	2,686,105
AST	9.00	91,390	36,190
APD	4.33	906,192	86,320
APD	3.00	10,518,300	100,992

*Correspondence concerning this article should be addressed to M.M. Faruque Hasan at hasan@tamu.edu, Tel.: 979-862-1449.

2 GCMC parameters for SOD Al substitution

2.1 Simulation parameters

The simulation parameters used for SOD for initial validation are provided here. Table S2 describes the force-field parameters from Garcia-Perez and table S3 shows the atom charges and descriptions used in the simulations.[1,2]. All non-bonded potentials are Lennard-Jones interactions.

Table S2: Force field interaction parameters for the Garcia-Perez force field[1,2]

Atom1	Atom2	$\epsilon/k_b(K)$	r (σÅ)
He	O	28.0	3.265
He	Oa	28.0	3.265
Ar	O	107.69	3.15
Ar	Oa	107.69	3.15
Ca	O	18.0	3.45
Ca	Oa	18.0	3.45
Na	O	23.0	3.4
Na	Oa	23.0	3.4
O_{co2}	O	84.93	2.9195
O_{co2}	Oa	84.93	2.9195
C_{co2}	O	50.2	2.7815
C_{co2}	Oa	50.2	2.7815
N_{n2}	O	58.25	3.062
N_{n2}	Oa	58.25	3.062
Na	CH_4^{sp3}	582.17	2.72
Na	CH_3^{sp3}	443.73	2.65
Na	CH_2^{sp3}	310.0	2.95
Na	CH^{sp3}	292.0	2.58
Na	C^{sp3}	126.0	2.71
Ca	CH_4^{sp3}	590.17	2.79
Ca	CH_3^{sp3}	400.00	2.6
Ca	CH_2^{sp3}	440.73	2.8
O	O_w	89.633	3.097
Oa	O_w	89.633	3.097
O_{co2}	O_{co2}	80.507	3.033
O_{co2}	C_{co2}	47.59	2.89
C_{co2}	C_{co2}	28.129	2.76
C_{benz}	O	89.00	3.007
C_{benz}	Oa	89.00	3.007
H_{benz}	O	71.24	2.606
H_{benz}	Oa	71.24	2.606
Na	C_{benz}	15.36439	3.545
Na	H_{benz}	14.56367	2.944
Na	C_{co2}	15.36439	3.545
Na	O_{co2}	14.56367	2.944

Table S3: Atom descriptions for GCMC simulations in SOD framework

**Name**	**Mass**	**Charge**
He	4.002602	0.0
Ar	39.948	0.0
O	15.9994	-1.025
Oa	15.9994	-1.2
Op	15.9994	-0.823
Si	28.0855	2.05
Al	26.981539	1.75
P	30.97376	2.35
Co	58.933195	0.0
Na	22.98977	1.0
Cl	35.4527	-1.0
C_{co2}	12.0	0.6512
O_{co2}	15.994	-0.3256

3 GCMC parameters for CHA Al substitution

The parameters for the CHA are different from those used for SOD and are provided in this section. The force field parameters from the Garcia Sanchez force field[5] are provided in Table S4. It is important to note that the oxygen atoms linked to Aluminum in the framework are identified differently in the force field. For each addition of Al, we add a Na cation for the charge balance. Table S5 shows the charge and radius of the atoms considered in the lattice. We assume that Si, Al, and O atoms as solid spheres.

Table S4: The non-bonded interaction parameters for GCMC force field parameters

Atom 1	Atom 2	$\epsilon/k_b(K)$	σ (Å)
Na	O	23.00	3.400
Na	O_a	23.00	3.400
Na	C_{CO_2}	362.29	3.320
Na	O_{CO_2}	200.83	2.760
O	C_{CO_2}	37.59	3.510
O	O_{CO_2}	78.98	3.237
O_a	C_{CO_2}	37.59	3.510
O_a	O_{CO_2}	78.98	3.237
O_{CO_2}	O_{CO_2}	85.67	3.017
C_{CO_2}	C_{CO_2}	29.99	2.745

Table S5: The list of atoms in the lattice. Data for their mass, charge, and radii are given.

Atom	Mass	Charge	Radii
O	15.9994	-0.39200	0.500
O_a	15.9994	-0.41384	0.500
Si	28.0855	0.78598	1.180
Al	26.981539	0.48598	1.180
C_{co2}	12.00	0.65120	0.720
O_{co2}	15.9994	-0.32560	0.680
Na	22.98977	0.38340	1.000

The location of cations can significantly affect the overall adsorption. However, fixing the position of ions requires a detailed analysis using density functional theory (DFT) [6]. To overcome this limitation, the Garcia Sanchez force field is used in this study, which allows the ions to move freely within the simulation domain. The parameters reported in their study use interaction parameters for free-floating cations in the lattice. However, the reinsertion of molecules in zeolite cages where it would not be physically possible due to channel and pore dimensions, due to diffusion, would occur. Thus, adsorption values using this approach would be slightly overestimated than experimentally obtained values.[7] Other factors such as imperfect crystals with defects may also lead to inconsistent values. This limitation of GCMC can be addressed by blocking certain cages in the framework to account for lattice defects but that is out of the scope of this paper.

Literature Cited

1. E Garcia-Perez, D Dubbeldam, Th LM Maesen, and Sofia Calero. Influence of cation na/ca ratio on adsorption in lta 5a: a systematic molecular simulation study of alkane chain length. *The Journal of Physical Chemistry B*, 110(47):23968–23976, 2006.

2. E García-Pérez, JB Parra, CO Ania, Antonio García-Sánchez, JM Van Baten, R Krishna, D Dubbeldam, and Sofia Calero. A computational study of co 2, n 2, and ch 4 adsorption in zeolites. *Adsorption*, 13:469–476, 2007.

3. Akhilesh Gandhi and MM Faruque Hasan. Machine learning for the design and discovery of zeolites and porous crystalline materials. *Current Opinion in Chemical Engineering*, 35:100739, 2022.

4. Akhilesh Gandhi and MM Faruque Hasan. A graph theoretic representation and analysis of zeolite frameworks. *Computers & Chemical Engineering*, 155:107548, 2021.

5. Almudena Garcia-Sanchez, Conchi O Ania, José B Parra, David Dubbeldam, Thijs JH Vlugt, Rajamani Krishna, and Sofia Calero. Transferable force field for carbon dioxide adsorption in zeolites. *The Journal of Physical Chemistry C*, 113(20):8814–8820, 2009.

6. Aleksei Vjunov, John L Fulton, Thomas Huthwelker, Sonia Pin, Donghai Mei, Gregory K Schenter, Niranjan Govind, Donald M Camaioni, Jian Zhi Hu, and Johannes A Lercher. Quantitatively probing the al distribution in zeolites. *Journal of the American Chemical Society*, 136(23):8296–8306, 2014.

7. Sajjad Ghojavand, Benoit Coasne, Edwin B Clatworthy, Rémy Guillet-Nicolas, Philippe Bazin, Marie Desmurs, Luis Jacobo Aguilera, Valérie Ruaux, and Svetlana Mintova. Alkali metal cations influence the CO_2 adsorption capacity of nanosized chabazite: modeling vs experiment. *ACS Applied Nano Materials*, 5(4):5578–5588, 2022.
